# Supramolecular Double-Helical Polymers: Supramolecular Chiral Induction and Asymmetric Catalysis

**DOI:** 10.3390/molecules30071517

**Published:** 2025-03-28

**Authors:** Xiaojun Guo, Xinyu Jia, Qin He, Wengui Duan, Yanjun Zhang, Yan Huang, Luzhi Liu

**Affiliations:** 1 School of Chemistry and Chemical Engineering, Guangxi University, Nanning 530004, China; kwok495@163.com (X.G.); 18715891782@163.com (X.J.); 13739016339@163.com (Q.H.); wgduan@gxu.edu.cn (W.D.); 2 Guangxi Key Laboratory of Green Chemical Materials and Safety Technology, Guangxi Engineering Research Center for New Chemical Materials and Safety Technology, College of Petroleum and Chemical Engineering, Beibu Gulf University, Qinzhou 535011, China; zhangyj201608@163.com; 3 Guangxi Key Laboratory of Traditional Chinese Medicine Quality Standards, Guangxi Institute of Chinese Traditional Medical & Pharmaceutical Science, Nanning 530022, China

**Keywords:** pillar[5]arenes, chiral induction, helical structure, asymmetric catalysis

## Abstract

Seeking a supramolecular chiral system induced by trace chiral molecules instead of traditional complex and expensive chiral ligands to achieve high yield or ee value conversion of the products is of great significance in asymmetric synthesis but still remains a challenge. Herein, two types of double helical supramolecular chiral systems, (*M*)-**Helix** and (*P*)-**Helix**, with opposite chiral optics were constructed in situ using tyrosine-functionalized pillar[5]arene as inducers. These systems exhibit chiroptical stability and enable remarkable chirality amplification from 7 mol% chiral seeds. When applied to intermolecular olefin cyano-trifluoromethylation, (*M*)-**Helix** exhibits remarkable catalytic efficiency (yield up to 89%), whereas (*P*)-**Helix** achieves higher enantioselectivity (ee up to 84%). This research will provide new ideas for supramolecular chiral catalysts in organic asymmetric catalysis applications.

## 1. Introduction

Asymmetric organometallic catalysis, a perfect combination of metal–organic chemistry and homogeneous catalysis, has made great progress in the last two decades [1,2,3]. However, due to the complexity of different optical chiral ligand preparations and the lack of regularity and adaptability of ligand–metal–substrate selective matching, it is challenging to construct a catalytic system that can accurately anticipate the selection of tailored enantiomers. It is therefore difficult to obtain two chiral ligands or catalysts that are complementary to the distinct stereoselectivity of the substrate, meaning that a high ee value enantiomer is gained while the other is lost, even if the latter is better suited to some specific biological activity or function. The development of simple and easily available chiral catalysts with complementary stereoselectivities for substrates to address the shortcomings of the aforementioned catalysts has become a goal that has been pursued by researchers [4,5]. The combination of supramolecular chemistry and catalysis has triggered a new field of supramolecular catalysis. Introducing chiral elements in a supramolecular manner and then constructing supramolecular chiral systems for catalytic reactions through non-covalent/covalent bonding have attracted much attention and made good progress due to the flexibility in design and avoidance of cumbersome synthesis [6,7,8]. For example, Nagata developed an (*M*)-**Helix** and a (*P*)-**Helix** containing poly (quinoline-2,3-diacetyl) side chains and diphenylphosphine pendants. The asymmetric hydrosilylation of styrene catalyzed by (*P*) or (*M*)-**Helix** and palladium yielded highly enantioselective (*R*)-product (94% ee) and (*S*)-product (90% ee), respectively [9]. Raynal developed the catalytic properties and structure of co-assemblies composed of a benzene-1,3,5-tricarboxamide (BTA) ligand coordinated to copper (the soldier) and seven enantiopure BTAs (the sergeants) have been determined; the catalytic system under study (58 % ee) [10]. However, the sensitive responsiveness of supramolecular chiral catalysts to external stimuli such as solvents and light significantly diminishes their supramolecular chiral stability and even causes chiral racemization or inversion, limiting the further application of the catalysts [11]. As a result, designing stable supramolecular chiral systems is one of the key issues in realizing highly stereochemical transformation control of substrates, which is also extremely challenging.

Supramolecular chiral systems are typically constructed by disrupting the symmetry during the nucleation process of self-assemblies through three primary strategies: (1) utilizing enantiopure monomers, (2) employing enantiopure reagents as chiral inducers, or (3) conducting polymerization in chiral solvents. Among these approaches, chiral inducers have gained significant attention due to their design flexibility, low required dosage, and ease of availability. Among them, chiral inducers are popular due to their design flexibility [12,13], low dosage, and easy availability [14,15,16]. Furthermore, self-assembly based on DNA-like double-helix supramolecular systems has been widely used in chemistry, materials science, and biomedicine due to its excellent chiroptical stability and controllability [17,18,19]. Inspired by the DNA double-helix architecture, the construction of highly active metal ions, such as Cu^+^, into analogous double-helix self-assembled systems is expected to result in stable macroscopic supramolecular chirality for asymmetric catalysis.

**Scheme 1:**
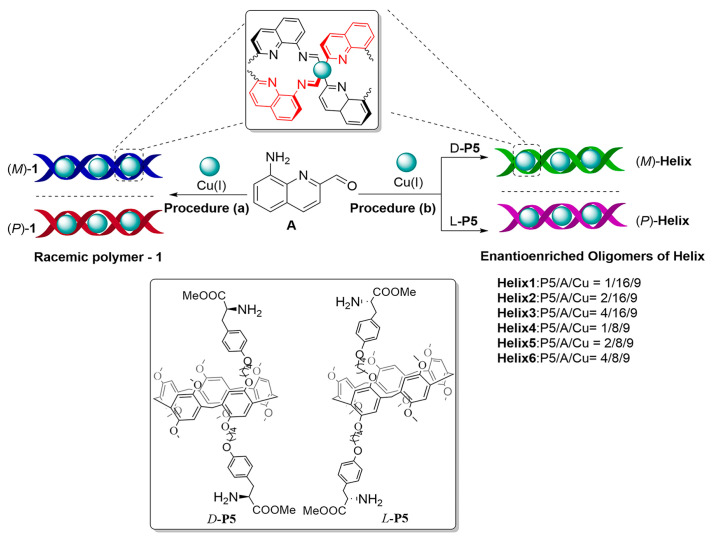
Self-assembly of double-helical racemic polymer 1 ^a^ and **Helix** ^b^ from monomers **A**. ^a^ Addition of monomer A to **Cu(I)** gave racemic helical polymer 1. ^b^ Introducing a chiral inducer, D-**P5** or L-**P5**, lent stereoselectivity to the polymerization reaction, yielding (*P*)-**Helix** or (*M*)-**Helix** preferentially.

Pillar[n]arenes [20,21,22,23] are a unique class of macrocyclic host compounds that have the intrinsic ability to create chirality by inhibiting the rotation of cavities through host-guest interaction or self-assembly, which is useful in the construction of chiral building blocks. Furthermore, the multi-action sites of pillar[n]arene are easily functionalized, and one or more self-assembly units can be tailored to specific requirements, providing not only flexible assembly strategies but also limitless possibilities for the creation of colorful supramolecular chiral systems [24,25,26]. As a result, pillar[n]arenes are the ideal materials for developing supramolecular chiral systems with specialized functions such as chiral recognition, chiral signal amplification, and chiral switching. To obtain stable supramolecular chirality, we attempted to construct a supramolecular catalytic system with a DNA-like double-helical structure (Scheme 1) by using D/L-tyrosine-functionalized pillar[5]arene D/L-**P5** as a chiral inducer, 8-amino-7-formylquinoline (**A**), and Cu(MeCN)_4_NTf_2_ containing cuprous ions **Cu(I)** as the reaction materials. The organic frameworks were first linked using dynamic covalent imine (C = N) bonds, and then the metal ions were incorporated into the framework through coordination of **Cu(I)** with the imine unit. Finally, a series of DNA-like double-helical supramolecular systems with different chirality, namely (*P*)-**Helix** and (*M*)-**Helix**, were constructed by chiral induction with chiral pillar[5]arene reagents. As expected, these systems have a pronounced optical chiroptical signal in the CD spectra while showing high yields and enantioselectivity in the intermolecular cyano-trifluoromethylation of olefins.

## 2. Results and Discussion

### 2.1. Self-Assembly and Characterization of Helix

**Figure 1:**
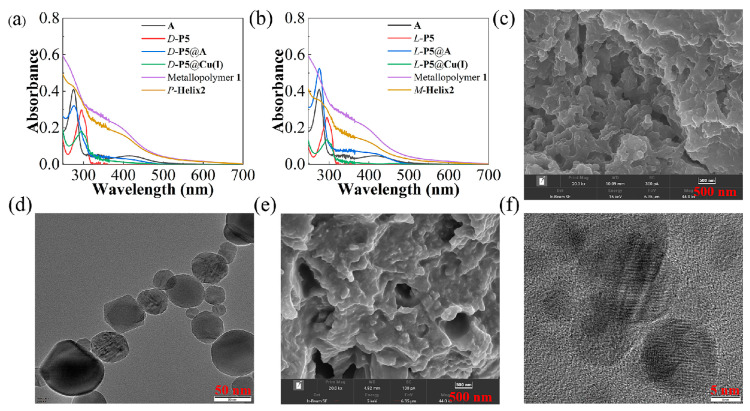
UV spectra of (**a**) (*P*)-**Helix2** and (**b**) (*M*)-**Helix2** in CH_3_CN. SEM (**c**) and TEM (**d**) of (*P*)-**Helix2**. SEM (**e**) and TEM (**f**) of (*M*)-**Helix2**.

Compounds D/L-**P5**, **A**, and Cu(MeCN)_4_NTf_2_ were synthesized and explicitly characterized by ^1^HNMR, ^13^CNMR, and HRMS (see the Supporting Information Figures S7–S19). The double helix polymer self-assembly strategy was shown in Scheme 1, in which two racemic helical polymers 1 (racemic-1) were obtained by adding **Cu(I)** to **A** in procedure (a). The procedure (b) introduced tyrosine-functionalized pillar[5]arene with different configurations, D-**P5** and L-**P5**, based on the procedure (a), which made the polymerization reaction stereoselective and led to the formation of chiral helical polymers. After the assembly of A with **Cu(I)** to form supramolecular racemic-1, the metal ligand charge transfer (MLCT) phenomenon occurred with significantly broader absorption peaks in the 250–500 nm range (Figure 1a) compared to the prereactive **A**. Similarly, (*P/M*)-**Helix** assembled using D/L-**P5** as chiral inducers also showed such MLCT behavior, which was attributed to the characteristic absorption of copper-containing supramolecular polymers.

**Figure 2:**
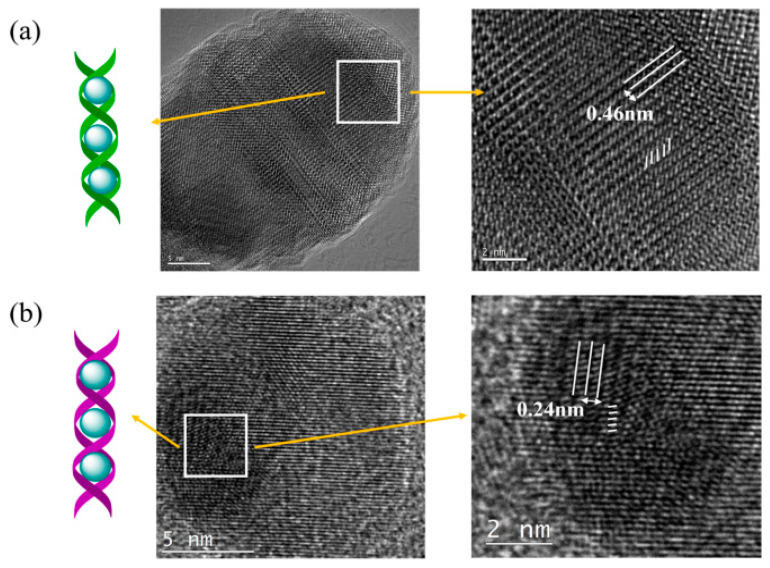
High-resolution TEM images of (**a**) (*P*)-**Helix2** and (**b**) (*M*)-**Helix2**; a series of striations are observable along the length of the aggregate; the polymers were well-defined, and a helical structure was observed at the structure (indicated by the white line).

**Figure 3:**
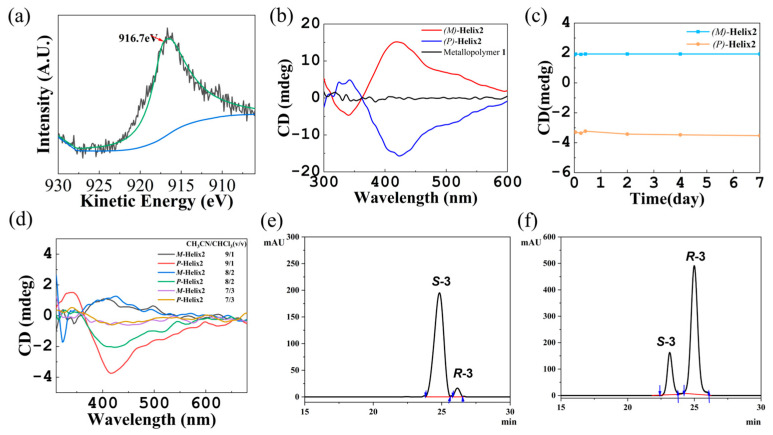
(**a**) X-ray photoelectron spectroscopy of (*P*)-**Helix2**. (**b**) CD spectra of (*P*)-**Helix2**, (*M*)-**Helix2**, and **Rac-2** in CH_3_CN. (**c**) Chiral stability of (*P*)-**Helix2** and (*M*)-**Helix2** in CH_3_CN. (**d**) CD spectra of (*P*)-**Helix2** and (*M*)-**Helix2** at different ratios of CH_3_CN/CHCl_3_ mixed solutions. High-performance liquid chromatography (HPLC) analysis of the enantioselectivity of (**e**) (*P*)-**Helix1** and (**f**) (*M*)-**Helix3** catalysts in trifluoromethyl cyanation of olefins.

Transmission electron microscopy (TEM) and scanning electron microscopy (SEM) revealed that (*P*)-**Helix2** and (*M*)*-***Helix2** could self-assemble into polygonal-shaped spheres and further aggregate into porous structures (Figure 1c–f). High-resolution TEM (Figure 2) clearly showed that parallel DNA-like double-helix structures were closely arranged and highly organized in the polygonal structure. This self-assembly mode is conducive to preserving the chirality and topology of the system. In addition, the principal binding energy of (*P*)-**Helix2** was 916.7 eV in X-ray photoelectron spectroscopy (Figure 3a), demonstrating that **Cu(I)** is the dominant form of copper in the supramolecular system [27,28,29].

### 2.2. Supramolecular Chirality

The formation of polymers (*P*)-**Helix2** and (*M*)-**Helix2** also promoted the generation of supramolecular chirality due to the presence of chiral inducers, as shown in Figure 3b. When enantiopure amine D-**P5** was used to nucleate the growth of a helical polymer from **A**, the polymer was found to have a preferred helical handedness, with a negative cotton effect at 325–375 nm and a positive broad cotton effect at 375–600 nm, which is remarkably comparable to the chiral system of (M)-**Helix** induced by (*R*)-4-aminobutane-1,2-diol^15^. That is, their supramolecular chirality resulted from the same inducing mechanism (amine induction): the D-**P5**-induced assembly promoted the development of (M)-type dominant helical polymers ((*M*)-**Helix**). In contrast, utilizing the amine’s other enantiomer, (*L*)-**P5**, produced the opposite helical chirality, similar to that induced by the (*S*)-4-aminobutane-1,2-diol, in that (*P*)-type helical polymers were the prevalent form ((*P*)-**Helix**). Unlike (*M*)-**Helix2** and (*P*)-**Helix2**, the CD signal of racemic polymer 1 was silent, which was attributed to the absence of enantiomeric bias in the system, forming a racemic helical metal polymer. These results fully demonstrated that D-**P5** and L-**P5** have excellent chiral induction properties, and supramolecular chiral systems with chiral inversion can be successfully obtained by regulating distinct chiral inducers.

Their self-assembly mechanism is depicted in Scheme 1. Compound A undergoes an addition–elimination reaction between aldehyde and amine groups, forming a polymer with dynamic covalent bonds (-C=N-). These dynamic covalent bonds coordinate with **Cu(I)** ions to form double-helical supramolecular structures. In the absence of a chiral environment, the double-helical supramolecules have an equal probability of polymerizing in a clockwise or counterclockwise direction, resulting in a racemic polymer (procedure a in Scheme 1). However, in the presence of a chiral inducer, the chiral pillar[5]arene amine forms a -C=N- bond with the aldehyde group of **A**. The spatial arrangement of these chiral inducers can influence the coordination mode of **Cu(I)** with the ligands and the polymer helicity, ultimately leading to the formation of enantiomerically enriched double-helical polymers (procedure b in Scheme 1). Although different enantiomerically chiral-induced helices embody mirrored chiral signals, their CD intensities (e.g., (M)-**Helix2** and (*P*)-**Helix2**) differ slightly, mainly due to the following factors: (1) slightly different enantiomeric excesses (ee) of the two systems; (2) challenges in maintaining consistency in the polymerization degree and ratios of the components of the two polymer systems; and (3) differences in the structural complexity and order of the helical arrangements. This phenomenon is similar to the chiral signal strength results reported for the enantiomerically chiral organic covalent framework CCOF by Yong Cui [30].

In order to further investigate the effect of chiral inducers on supramolecular chirality, the chiral inducing properties of the systems with different contents of tyrosine-based pillar[5]arene were investigated. It was found that the supramolecular chirality of the system was largely dependent on the chiral inducer. Interestingly, the strength of the chiral signal was not linearly related to the amount of chiral inducer added (see the Supporting Information Figure S21), which could be primarily attributable to the following reasons: (1) Helix formation requires a ratio of compound **A** to Cu^+^ of 2:1; an excess of P5 interferes with this interaction by generating imine bonds. (2) The large size of P5 affects self-assembly, altering the topology and particle size and thus the chirality. The supramolecular chirality of the system was optimized when the molar fraction of the chiral inducer was 7% (**P5**/**A**/**Cu(I)** = 2/16/9, (*P/M*)-**Helix2**).

Furthermore, the supramolecular chiral behavior of the (*P*)-**Helix2** and (*M*)-**Helix2** systems in various solvents was investigated. They had the best CD signals in CH_3_CN solvent, and the signal remained stable even after one week of continuous observation (Figure 3c), which may be due to the double helical structure formed by the incorporation of the metal ions to stabilize the chirality. However, the supramolecular chirality of the system was greatly affected by solvents. The CD signals at 325–375 nm were faint or barely detectable in DMSO, DMF, and CHCl_3_ (see the Supporting Information Figure S22). In a mixed solution of CH_3_CN/CHCl_3_, the CD signals of (*P*)-**Helix2** and (*M*)-**Helix2** diminished with the continuous addition of CHCl_3_ solvent and were almost completely suppressed at CH_3_CN/CHCl_3_ (7/3). The main reason was that the difference in CD signal was caused by the influence of different solvents on the ratio of (*P*)-**Helix** and (*M*)-**Helix** in the system. Solvent polarities can affect van der Waals interactions, including π–π stacking between molecules. Furthermore, these solvents (DMSO, DMF, and CHCl_3_) possess strong ligand groups or multiple binding sites that interact with copper ions, affecting the coordination ability and mode of ligand A with copper. These factors can lead to geometrical instability, depolymerization, or rearrangement of chiral supramolecules. In contrast, CH_3_CN, which has limited N-coordination capacity, interacts with copper ions without appreciably modifying the polymer’s structure, allowing it to preserve its chirality and be suited for further asymmetric catalysis.

### 2.3. Asymmetric Catalysis

Incorporation of trifluoromethyl (CF_3_) substituents into small molecules has become an important functional group for modulating the physical properties of new drugs due to their excellent metabolic stability and lipophilicity, as well as electron adsorption [31,32,33,34]. Asymmetric trifluoromethylation of olefins, particularly inactivated olefins, has emerged as a research hotspot in recent years, with successful approaches being extensively researched [35,36,37,38]. However, the catalysts or ligands utilized are costly and require extensive preparation procedures. Our constructed DNA-like double helical supramolecular polymer had good chiral amplification capability and stability in the reaction solvent CH_3_CN. This allowed it to effectively regulate the asymmetric environment surrounding the metal center and satisfy the requirements of asymmetric catalysis. Therefore, (*P/M)*-**Helix** was chosen as a supramolecular catalyst instead of a traditional copper catalyst to study the enantioselectivity of cyanotrifluoromethylated styrenes.

The study focused on the reaction of styrene (4) with Togni’s reagent (5) in the presence of a copper catalyst and using trimethylcyanosilane (TMSCN) as a cyano source. Considering the superior supramolecular chirality of Helix2, the reaction was initially carried out using the (*M*)-**Helix2** catalyst to examine the synthetic yield (see the Supporting Information Table S2). After extensive screening of the reaction parameters, we discovered that the cyanotrifluoromethylation reactions of olefins at room temperature in the presence of (*M*)-**Helix2** (5.0 mol%) in CH_3_CN gave the best results, affording the desired product 3 in 89% isolated yield. Notably, the reaction efficiency appeared to be sensitive to the solvents. For instance, the use of DMSO, DMF, or CHCl_3_ gave just traces of the desired product 3. Following that, the self-assembled supramolecular catalysts with various copper ratios were tested for catalytic yields and enantioselectivity (Table 1). All the produced supramolecular catalysts displayed specific yields and ee values, with the maximum yield obtained at a copper molar ratio **A**/**Cu(I)** of 16/9. However, the yield declined dramatically as the copper molar fraction increased. Overall, the (*M*)-**Helix** catalyst was more favorable for product conversion, yielding up to 89%. The (*P*)-**Helix** type catalyst had higher enantioselectivity, with an ee value of up to 84%.

Surprisingly, the stereoselectivity of the supramolecular catalysts did not depend entirely on the concentration of the chiral inducer. Among (*P*)-**Helices**, (*P*)-**Helix1** exhibited superior stereoselectivity despite having the lowest molar fraction of the chiral inducer (4%). In contrast, among (*M*)-**Helices**, (*M*)-**Helix3** demonstrated excellent stereoselectivity with the highest molar fraction of the chiral inducer (14%). (*R*)-**3** and (*S*)-**3** were effectively separated in high-performance liquid chromatography (HPLC) using a mobile phase of Isopropyl alcohol/hexane = 40/1, a flow rate of 0.7 mL/min, and a chiral column of OD-H. The baselines for both enantiomers were completely resolved with longer retention times observed for (*R*)-**3** (Figure 3e,f), consistent with previous findings in Liu^33^ reported. Specifically, it was determined that (*P*)-**Helix1** predominantly catalyzed the formation of the (*S*) configuration as an enantiomeric product, whereas (*M*)-**Helix3** favored catalysis toward the formation of the (*R*) configuration.

The great difference in yield and enantioselectivity between (*P*)-**Helix** and (*M*)-**Helix** in cyanotrifluoromethylation of styrene prompted further investigation of their topologies using **Helix2** as templates. HRTEM (Figure 2) and AFM (Figures S29 and S30) revealed irregular polygonal morphologies for both polymers. (*M*)-**Helix** exhibited larger surface roughness (34.3 nm) and surface area (1.72 μm^2^) compared to (*M*)-**Helix**, which likely contributed to its better catalytic efficiency. Generally speaking, the larger the catalytic surface area, the more effective the adsorption of reactant molecules, which promoted the catalytic activity and increased the reaction yield [39,40]. Regarding enantioselectivity, the proximity between the asymmetric environment of Togni reagent 2 and the Cu ion center of the supramolecular catalyst would play an important role in the stereoconversion of the substrate. HRSEM revealed a smaller helical spacing in (*M*)-**Helix** (0.24 nm), which was not conducive to the spatial tuning of the activation site of compound 2 to the Cu ion’s asymmetric environment, reducing stereoselectivity. In contrast, the larger helical spacing in (*M*)-**Helix** (0.36 nm) allowed more room for the Togni reagent to adapt during activation, facilitating better stereoselectivity regulation.

## 3. Materials and Methods

### 3.1. General

^1^H and ^13^C NMR spectra were recorded on a Brucker AV600 MHz spectrometer (Billerica, MA, USA). FT-IR spectra were recorded on a Nicolet (Quebec, QC, USA) iS 50 FT-IR. UV/Vis spectra and the optical transmittance were recorded on a quartz cell (light path 10 mm) on a Shimadzu (Tokyo, Japan) UV-1800 spectrophotometer. High-resolution transmission electron microscopy (TEM) images were acquired using a Tecnai (Hillsboro, OR, USA) 20 high-resolution transmission electron microscope operating at an accelerating voltage of 200 keV. The sample for high-resolution TEM measurements was prepared by dropping the solution onto a copper grid. The grid was then air-dried. Scanning electron microscope (SEM) images were recorded on a TESCAN (Brno, Czech Republic) MIRA LMS. X-ray photoelectron spectroscopy (XPS) tests were performed by a Thermo Scientific (Waltham, MA, USA) K-Alpha analysis system. Atomic force microscope (AFM) images were recorded on a Bruker Dimension Icon. Circular dichroism (CD) spectroscopy tests were performed by a MOS-450 (Bio-logic, Seyssinet-Pariset, France). High-performance liquid chromatography (HPLC) tests were performed by a UPLCI-CLASS-XEVOG2-XSQTOF(Waters, Xevo G2-XS QTof, Milford, MA, USA). Other reagents were purchased from commercial suppliers and used as received.

### 3.2. Synthesis of A and Tyrosine-Functionalized Pillar[5]arene P5

Compounds A and P5 were synthesized according to existing procedures [17,41], and can be found in the Supplementary Information.

**A**. White solid; yield: 70%; melting point: 72–73 °C; IR (KBr) cm^−1^: 3367.35 (N-H), 1718.37 (C=O), 1606.12 (C=C), 1518.37, 1489.80 (ArC=C), 1569.39 (C=N), 1014.29 (C-O); ^1^H NMR (600 MHz, Chloroform-d) δ 9.05 (s, 1H), 8.37 (d, *J* = 7.6 Hz, 1H), 8.00 (d, *J* = 8.4 Hz, 1H), 7.43 (t, *J* = 7.9 Hz, 1H), 7.36 (dd, *J* = 8.2, 1.3 Hz, 1H), 7.29 (d, *J* = 8.4 Hz, 1H), 2.73 (s, 3H), 1.59 (s, 9H). ^13^C NMR (126 MHz, Chloroform-d) δ 156.97, 152.99, 137.58, 136.31, 134.57, 126.21 (d, *J* = 17.2 Hz), 122.33, 119.93, 114.35, 80.34, 28.45, 27.43, 25.20.

**P5**. White solid; yield: 80%; melting point: 135.5–164.4 °C; D-**P5** = −67.1838; IR (KBr) cm^−1^: 3424.05 (N-H), 2937.58, 2851.77 (C–H), 1501.34 (Ar–C = C), 1212.06, 1046.69 (Ar–O, C–O); ^1^H NMR (600 MHz, Chloroform-d) δ 7.10 (d, *J* = 8.6 Hz, 4H), 6.84 (d, *J* = 8.5 Hz, 4H), 6.79–6.75 (m, 10H), 4.00 (t, *J* = 5.8 Hz, 4H), 3.88 (m, 7H), 3.79–3.62 (m, 41H), 3.04 (dd, *J* = 9.4 Hz, 2H), 2.83 (dd, *J* = 10.8 Hz, 2H), 1.81 (s, 8H); ^13^C NMR (151 MHz, Chloroform-d) δ 168.50, 151.44, 150.27, 140.88, 140.82, 130.24, 128.98, 127.59, 121.07, 119.18, 114.55, 113.62, 67.43, 56.15, 55.57, 51.95, 47.48, 44.24, 43.94, 42.48, 40.68, 40.16, 29.85, 29.81, 29.74, 29.71, 29.67, 29.63, 26.29, 26.08; HRESI–MS *m*/*z*: 1221.58923 [M + H^+^] (Calcd for C_71_H_85_N_2_O_16_, 1221.5894).

### 3.3. Typical Self-Assembly Procedure of Helix2

Under an N_2_ atmosphere, monomer **A** (5 mg, 0.030 mmol, 16 eq), **[Cu]** (9.68 mg, 0.015 mmol, 9 eq), and **P5** (4.88 mg, 10 mg/mL dissolved in CH_3_CN, 3.75 umol, 2 eq) were added to dry, degassed CH_3_CN (0.5 mL) to give a brown solution, which was stirred at room temperature for 48 h. The solvent was evaporated to give a dark brown solid. Self-assembled supramolecular helical polymers with different **P5**/**A**/**Cu(I)** molar ratios were synthesized according to the same procedure.

### 3.4. Trifluoromethylation of Styrene

Styrene 4 was selected as the model substrate to optimize the reaction conditions. The study focused on the reaction of styrene with Togni’s reagent, 3,3-dimethyl-1-(trifluoromethyl)-1,2-benzoiodoxacyclo, in the presence of a copper catalyst, and trimethylcyanosilane (TMSCN) was used as the cyano source. Initially, the catalyst used for the reaction was a *P*-P2 supramolecular handifold system assembled by 2 eq L-**PCSA**, 16 eq **A**, and 9 eq **[Cu]**, and the reaction was carried out in acetonitrile solution at 60 °C with trimethylcyanosilane (1.5 eq) and Togni reagent 3,3-dimethyl-1-(trifluoromethyl)-1,2-benzoiodoxacyclo (1.2 eq) in the presence of 10 mol% (*P*)-**P2** and (*M*)-**P2** for 12 h, respectively. On this basis, the conditions are optimized.

## 4. Conclusions

In summary, inspired by the double-stranded DNA helix, we have successfully constructed a class of supramolecular metallopolymers with double helixes, which have stable and good supramolecular chirality. The stable DNA-like double-helical supramolecular chiral system constructed by trace amounts of chiral molecules can obtain good asymmetric catalytic selectivity. The asymmetric catalysis of intermolecular cyano-trifluoromethylation of olefins had excellent performance, with yields as high as 89% for (*M*)-Helix2 and 84% enantioselectivities for (*P*)-Helix1. Interestingly, the enantioselective conversion of the substrate does not depend exclusively on the chiral strength of the supramolecular catalyst. The selection of supramolecular chiral catalysts should be examined in various dimensions rather than focusing only on the chiral strength of the supramolecules. This study will provide new ideas for supramolecular chiral catalysts in organic asymmetric catalysis applications.

## Figures and Tables

**Table 1:** The yields and enantioselectivitie of product 3 in different supramolecular catalysts.

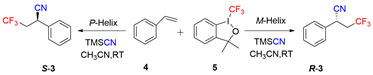			
Entry	Supramolecular Catalyst	Yield (%)	ee(%): (R − S)/(R + S)
**1**	(*P*)-**Helix1**	39	85 (*R*)
**2**	(*M*)*-***Helix1**	84	12 (*S*)
**3**	(*P*)*-***Helix2**	35	28 (*R*)
**4**	(*M*)*-***Helix2**	89	3 (*S*)
**5**	(*P*)*-***Helix3**	20	21 (*R*)
**6**	(*M*)*-***Helix3**	70	59 (*S*)
**7**	*Rac*-**1**	85	<2% (*R*)

## Data Availability

The data presented in this study are available in this manuscript.

## Supplementary Materials

The following supporting information can be downloaded at https://www.mdpi.com/article/10.3390/molecules30071517/s1.
