# Supplementary material for: Supramolecular Double-Helical Polymers: Supramolecular Chiral Induction and Asymmetric Catalysis
Source: Molecules. 2025 Mar 28;30(7):1517. doi: 10.3390/molecules30071517 (PMC11990125; doi:10.3390/molecules30071517)
Supplement: Supplementary file 1 [file molecules-30-01517-s001.zip › molecules-3500858-supplementary.pdf]

Supporting Information

DNA-like supramolecular double-helical polymers: supramolecular chiral induction and asymmetric catalysis

Xiaojun Guo†,Xinyu Jia†, Qin He†, Wengui Duan†, Yanjun Zhang†, Yan Huang‡, and Luzhi Liu†*

† Guangxi Colleges and Universities Key Laboratory of Applied Chemistry Technology and Resource Development, School of Chemistry and Chemical Engineering, Guangxi University, Nanning 530004, Guangxi, P. R. China , Guangxi Key Lab of Agricultural Resources Chemistry and Biotechnology, College of Chemistry and Food Science, Yulin Normal University, Yulin, Guangxi 537000, PR China Email: llzh068@163.com

‡Guangxi Institute of Chinese Traditional Medical & Pharmaceutical Science and Guangxi Key Laboratory of Traditional Chinese Medicine Quality Standards, Nanning 530022, Guangxi, P. R. China

Contents

1. Materials and methods 2

2. Synthesis of P5 3

3. Synthesis of A 12

4. Synthesis of Cu(I) 18

5. Typical self-assembly procedure 20

6. CD spectra 22

7. Asymmetric catalytic reactions with supramolecular catalysts 23

8. HPLC of the Products 26

9. AFM of Helix 30

10. Reference 28

1. Materials and methods

Materials

All reagents were commercially available and used as supplied without further purification. Solvents were either employed as purchased or dried according to procedures described in the literature.

Measurements

^{1}H and ^{13}C NMR spectra were recorded on a Brucker AV600 MHz spectrometer. FT-IR spectra were recorded on a Nicolet iS 50 FT-IR. UV/Vis spectra and the optical transmittance were recorded on a quartz cell (light path 10 mm) on a Shimadzu UV-1800 spectrophotometer. High-resolution Transmission electron microscopy (TEM) images were acquired using a Tecnai 20 high-resolution transmission electron microscope operating at an accelerating voltage of 200 keV. The sample for high-resolution TEM measurements was prepared by dropping the solution onto a copper grid. The grid was then air-dried. Scanning Electron Microscope (SEM) images were recorded on a TESCAN MIRA LMS. X- ray photoelectron spectroscopy (XPS) tests were performed by a Thermo Scientific K-Alpha analysis system. Atomic Force Microscope (AFM) images were recorded on a Bruker Dimension Icon. Circular Dichroism (CD) spectroscopy tests were performed by a MOS-450. High Performance Liquid Chromatography (HPLC) tests were performed by a UPLCI-CLASS-XEVOG2-XSQTOF

2. Synthesis of P5

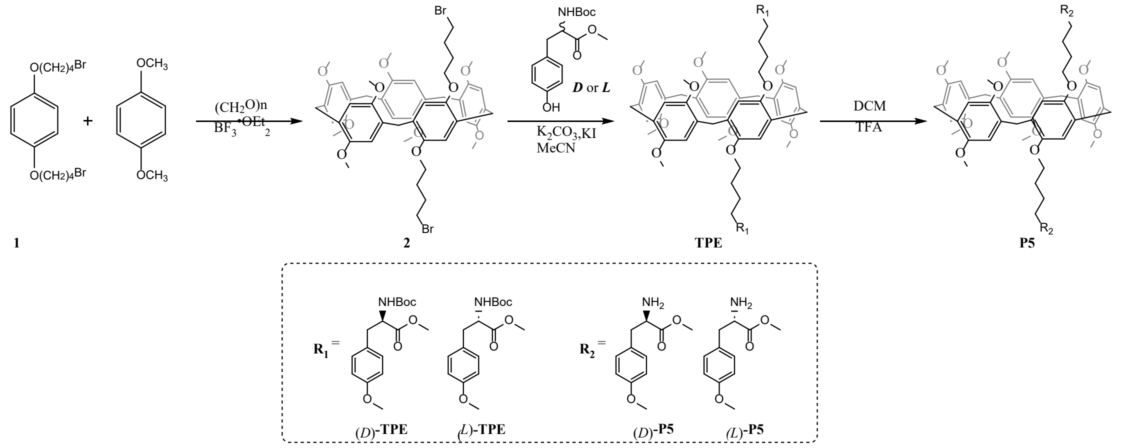

Scheme *S*1. Synthetic route of **P5**

Compound **1** was synthesized according to the literature[1]. To a solution of compound **1** (2.00g, 5.26mmol) and 1,4-dimethoxybenzene (2.91g, 21.05mmol) in $ClCH_2CH_2Cl$ (80ml), paraformaldehyde (0.79g, 26.31mmol) was added at 30℃. Boron trifluoride diethyl etherate (7.10mL, 26.31mmol) was then added to the solution and the mixture was stirred at room temperature for 2h and concentrated by rotary evaporation. The resultant oil was dissolved in CH_2Cl_2 and washed twice with H_2O. The organic layer was dried over anhydrous Na_2SO_4 and evaporated to afford the crude product, which was isolated by flash column chromatography using dichloromethane/petroleum ether (DCM/PE =1/1, v/v) to give compound **2** (1.56g, 29.8%) as a white solid.

Compound **2** (600mg, 0.60mmol), D- or L-type of N-*tert*-Butoxycarbonyl tyrosine methyl ester (892mg, 3.02mmol), K_2CO_3 (835mg, 6.04mmol) and KI (501mg, 3.02mmol) were added to acetonitrile (25ml) solution to prepare compound **TPE**, which reacted at 75 ℃ for 12 h. At the end of the reaction,

deionized water (100ml) was added and the product was extracted with CH_2Cl_2 (3×40ml). The organic phase was collected and concentrated under reduced pressure. After column chromatography (silica gel, EA/PE=10/1, v/v), pale yellow oily liquid was obtained. After vacuum distillation, white solid (78.5%) product D-**TPE** and L-**TPE** was obtained.

D-**TPE** melting point:71.8-75.1℃; IR (KBr) cm^{-1}: 3437.29 (N-H), 2927.24, 2849.91 (C–H), 1717.92 (C=O), 1612.61 (N-H), 1499.09, 1461.24 (Ar–C=C), 1212.80, 1049.91 (Ar–O, C–O); ^{1}H NMR (600 MHz, Chloroformd) δ 7.03 (d, J =8.4 Hz, 4H), 6.83 (d, J =8.6 Hz, 4H), 6.81-6.72 (m, 10H), 4.98 (d, J =8.0 Hz, 2H), 4.55 (q, J =5.9 Hz, 2H), 4.09–3.56 (m, 48H), 3.03 (ddt, J =19.6, 14.0, 5.8 Hz, 4H), 2.10 – 1.87 (m, 8H), 1.43 (s, 18H);^{13}C NMR (151 MHz, Chloroform-d) δ 172.58, 150.98, 150.96, 150.90, 150.86, 150.11, 130.43, 128.47, 128.44, 128.42, 128.33, 128.24, 115.09, 114.62, 114.35, 114.18, 114.11,68.13, 67.63, 55.96, 55.94, 55.90, 54.69, 52.33, 37.60, 29.85, 29.74, 26.65, 26.48; ESI–MS m/z:$C_{81}H_{100}N_2O_{20}$: 1438.70349 ([M+NH_4^+]).

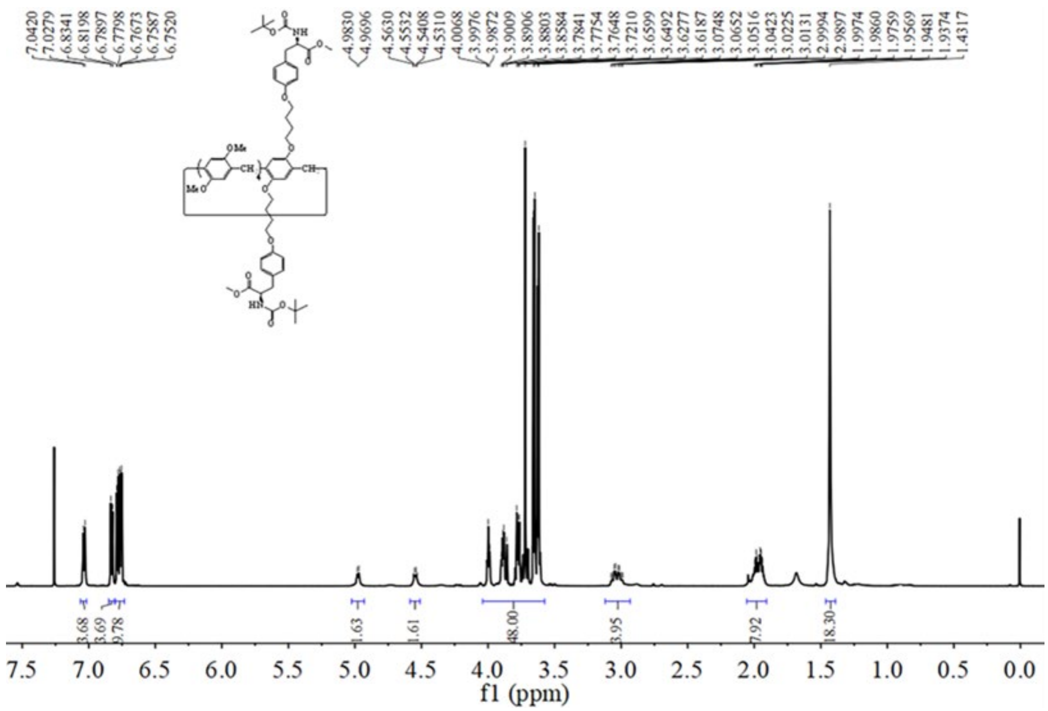

Figure S1. ^{1}H NMR spectrum of D-**TPE** in $CDCl_3$.

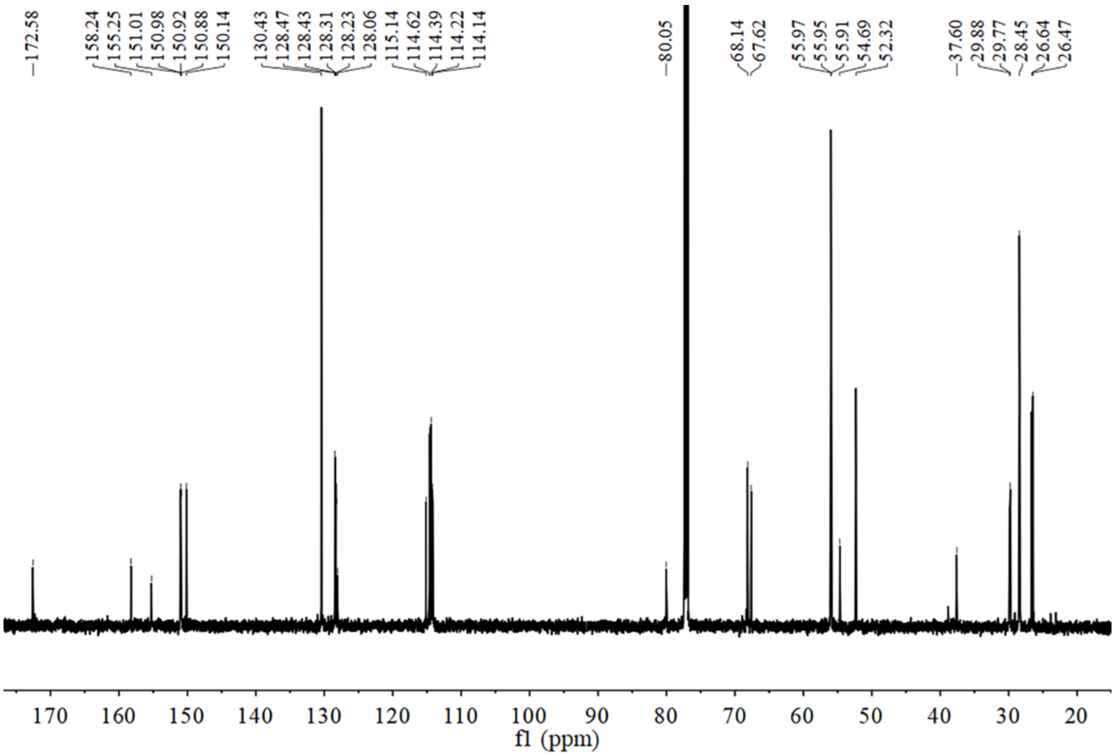

Figure S2. ^{13}C NMR spectrum of D-**TPE** in $CDCl_3$.

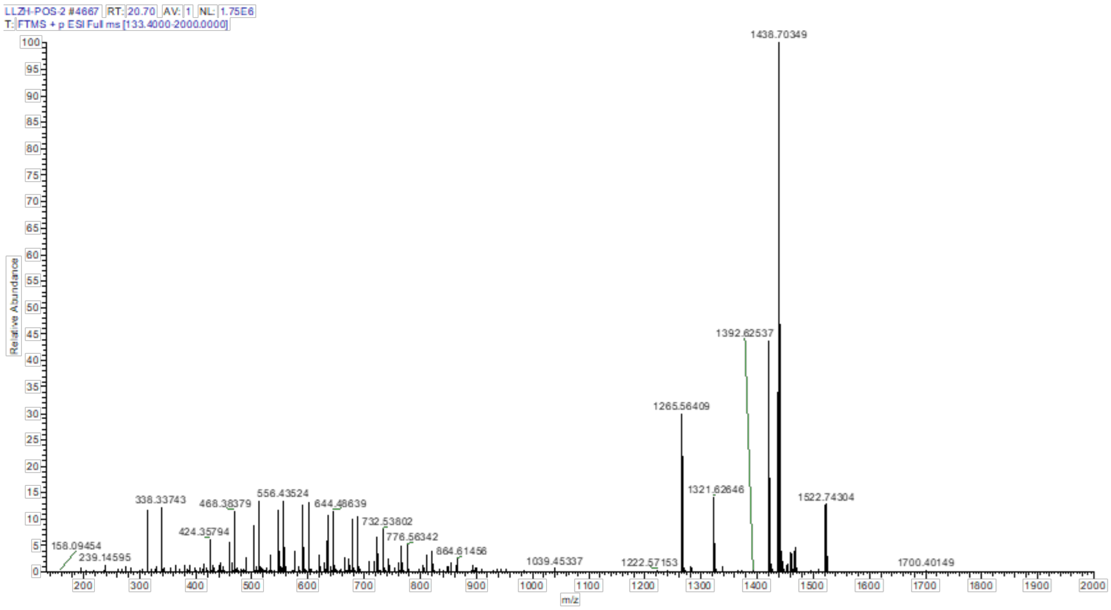

Figure S3. ESI-MS spectrum of D-**TPE**.

L-**TPE** melting point: 71.8-75.2℃; IR (KBr) cm^{-1}: 3434.00 (N-H), 2932.18, 2851.55 (C–H),1719.56 (C=O), 1609.32 (νN-H), 1499.78, 1462.89 (Ar–C=C), 1209.51, 1051.55 (Ar–O, C–O); 1H NMR (600 MHz, Chloroform-d) δ 7.03 (d,J =8.4 Hz, 4H), 6.83 (d, J =8.6 Hz, 4H), 6.81 – 6.72 (m, 10H), 4.98 (d, J =8.0 Hz, 2H), 4.55 (q, J =5.9 Hz, 2H), 4.09 – 3.56 (m, 48H), 3.03 (ddt, J =19.6, 14.0, 5.8 Hz, 4H), 2.10 – 1.87 (m, 8H), 1.43 (s, 18H); ^{13}C NMR (151 MHz, Chloroform-d) δ 172.58, 150.98, 150.96, 150.90, 150.86, 150.11, 130.43, 128.47, 128.44, 128.42, 128.33, 128.24, 115.09, 114.62,114.35, 114.18, 114.11, 68.13, 67.63, 55.96, 55.94, 55.90, 54.69, 52.33, 37.60, 29.85, 29.74, 26.65,26.48; HRESI–MS m/z: 1438.70349 $[M+H_2O^+]$ (Calcd for $C_{81}H_{104}N_3O_{20}$, 1438.6970).

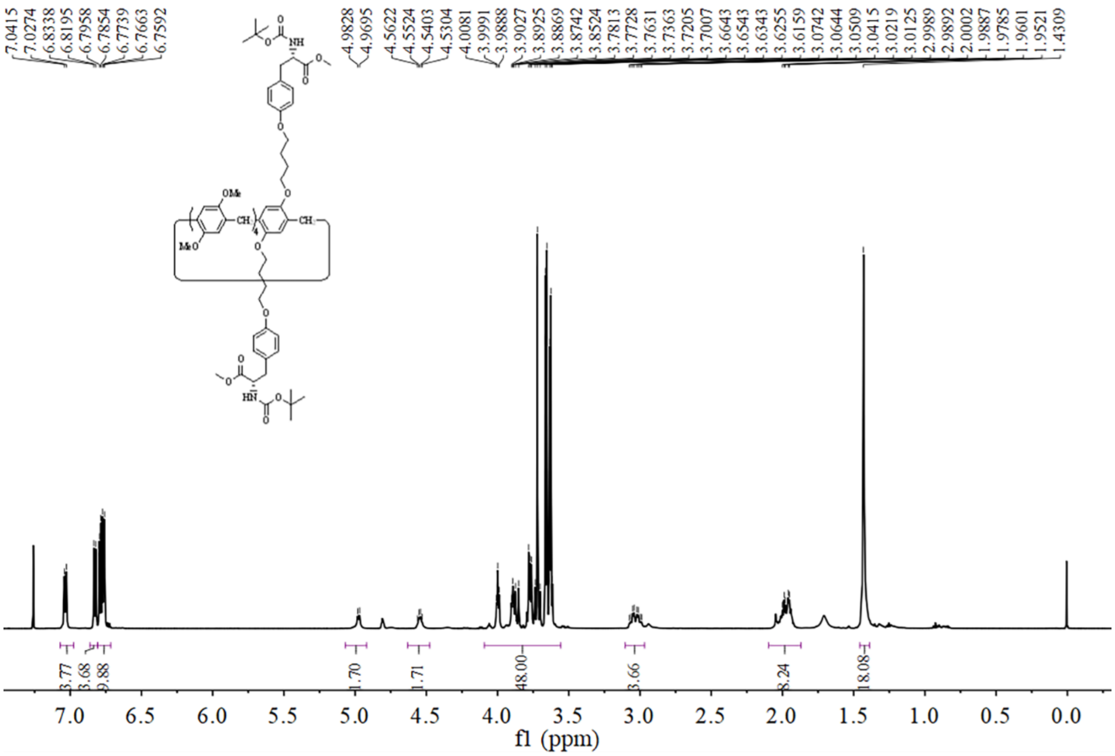

Figure S4. ^{1}H NMR spectrum of L-**TPE** in $CDCl_3$.

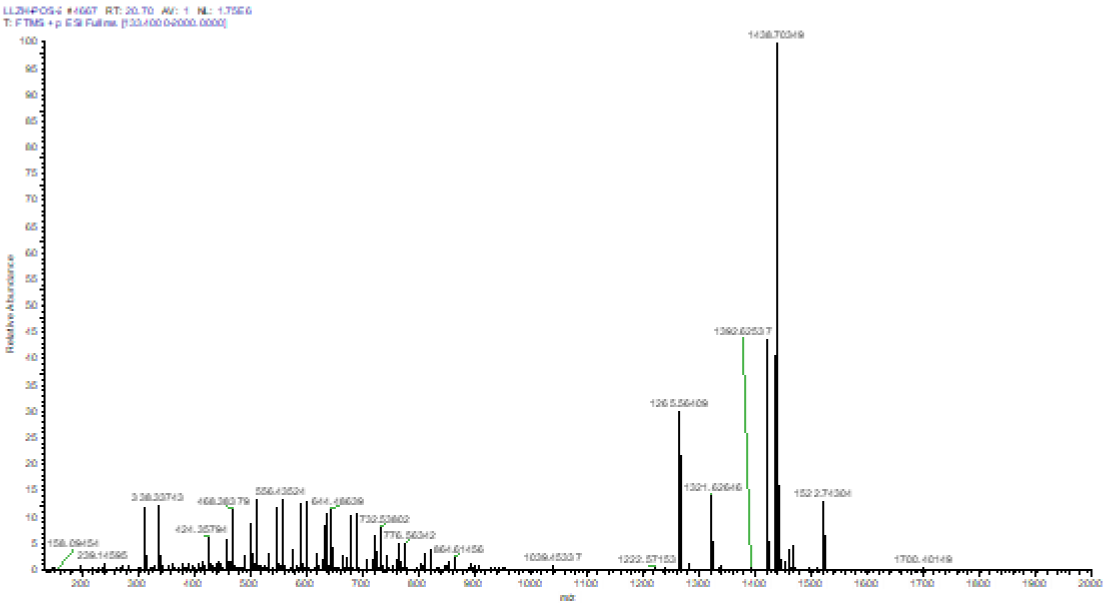

Figure S5. HRESI-MS spectrum of L-**TPE** in $CDCl_3$.

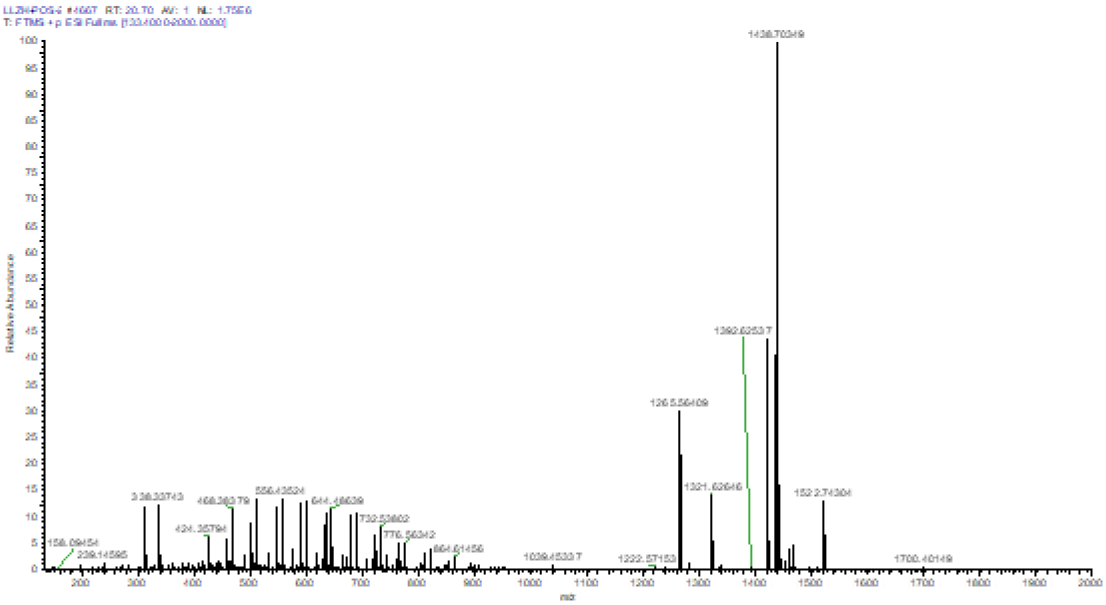

Figure S6. HRESI-MS spectrum of L-**TPE**.

100 mg of the compound D-**TPE** or L-**TPE**, 3mg of trifluoroacetic acid, and 8mL of dichloromethane were added in a 50ml two-port flask. Stir well and react at 50℃ for 1h, during which TCL is used to track the reaction process. Add 500 mL deionized water, 10g $NaHCO_3$, 0.186g EDTA, heat and boil, add into dialysis bag and soak 10mim. Heat 500mL deionized water, add 0.186g EDTA, transfer dialysis bag and soak for 10min. The reaction mixture was put into a dialysis bag and soaked in 500ml deionized water for 0.5h, 4h and 6h, respectively, during which TCL tracked the reaction process and changed the water, and vacuum dried to obtain D or L-**P5** with white solid (80.3%,80.3%).

D-**P5** melting point: 135.5-164.4℃; $[\alpha]_{D\text{-}P5}$ =-67.1838; IR (KBr) cm^{-1}:3424.05 (N-H), 2937.58, 2851.77 (C–H), 1501.34 (Ar–C=C), 1212.06, 1046.69 (Ar–O,C–O); ^{1}H NMR (600 MHz, Chloroform-d) δ 7.10 (d, J =8.6Hz, 4H), 6.84 (d, J=8.5 Hz, 4H), 6.79–6.75 (m, 10H), 4.00 (t, J =5.8 Hz, 4H), 3.88 (m,7H), 3.79–3.62 (m, 41H), 3.04 (dd, J=9.4Hz, 2H), 2.83 (dd, J=10.8 Hz,2H), 1.81 (s, 8H); ^{13}C NMR (151 MHz, Chloroform-d) δ 168.50, 151.44, 150.27, 140.88, 140.82, 130.24, 128.98, 127.59, 121.07, 119.18, 114.55, 113.62, 67.43, 56.15, 55.57, 51.95, 47.48, 44.24, 43.94, 42.48, 40.68, 40.16, 29.85, 29.81, 29.74, 29.71, 29.67, 29.63, 26.29, 26.08; HRESI–MS m/z: 1221.58923 $[M+H^{+}]$ (Calcd for $C_{71}H_{85}N_{2}O_{16}$, 1221.5894).

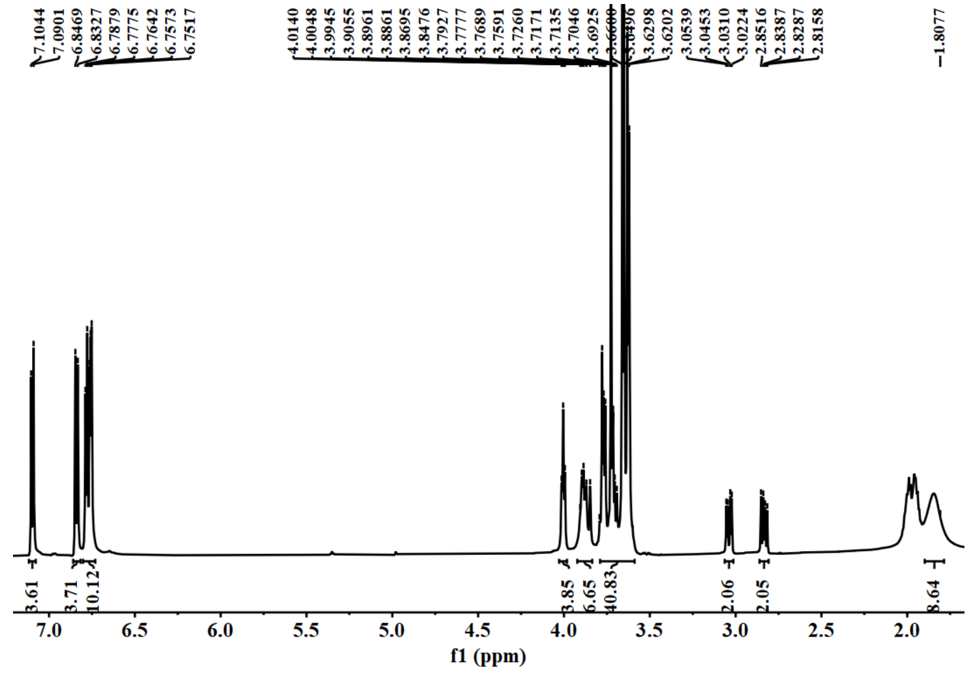

Figure S7. ^{1}H NMR spectrum of D-**P5** in $CDCl_3$.

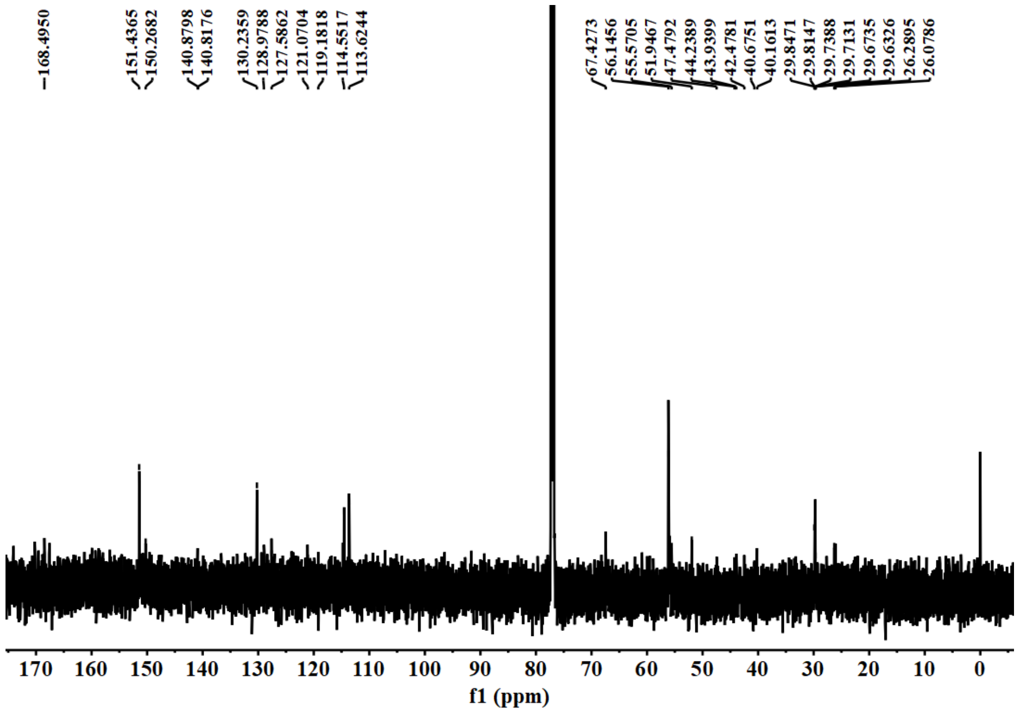

Figure S8. ^{13}C NMR spectrum of D-**P5** in $CDCl_3$.

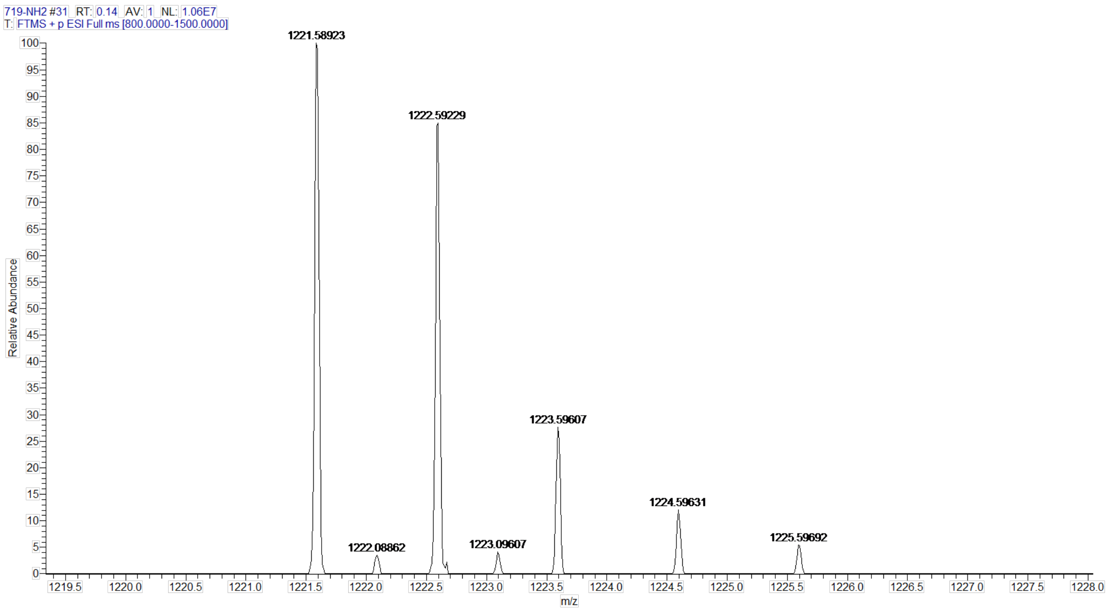

Figure S9. HRESI-MS spectrum of D-**P5**.

L-**P5** melting point:134.4-141.6℃; $[\alpha]_{L\text{-}P5}$=36.8400; IR (KBr) cm^{-1}: 3424.05 (N-H), 2937.58, 2851.77 (C–H), 1501.34 (Ar–C=C), 1212.06, 1046.69 (Ar–O, C–O); ^{1}H NMR (600 MHz, Chloroform-d) δ 7.10 (d, *J* =8.6 Hz, 4H), 6.84 (d, J =8.5 Hz, 4H), 6.79 – 6.75 (m, 10H), 4.00(t, *J* =5.8 Hz, 4H), 3.88 (m, 7H), 3.79 – 3.62 (m, 41H), 3.04 (dd, J =9.4Hz, 2H), 2.83 (dd, *J* =10.8 Hz, 2H), 1.81 (s, 8H); ^{13}C NMR (151 MHz, Chloroform-d) δ168.50, 151.44, 150.27, 140.88, 140.82, 130.24, 128.98, 127.59, 121.07, 119.18, 114.55, 113.62, 67.43, 56.15, 55.57, 51.95, 47.48, 44.24, 43.94, 42.48, 40.68, 40.16, 29.85, 29.81, 29.74, 29.71,29.67,29.63,26.29,26.08;ESI–MS m/z: 1221.58923 $[M+H^{+}]$ (Calcd for $C_{71}H_{85}N_{2}O_{16}$, 1221.5894).

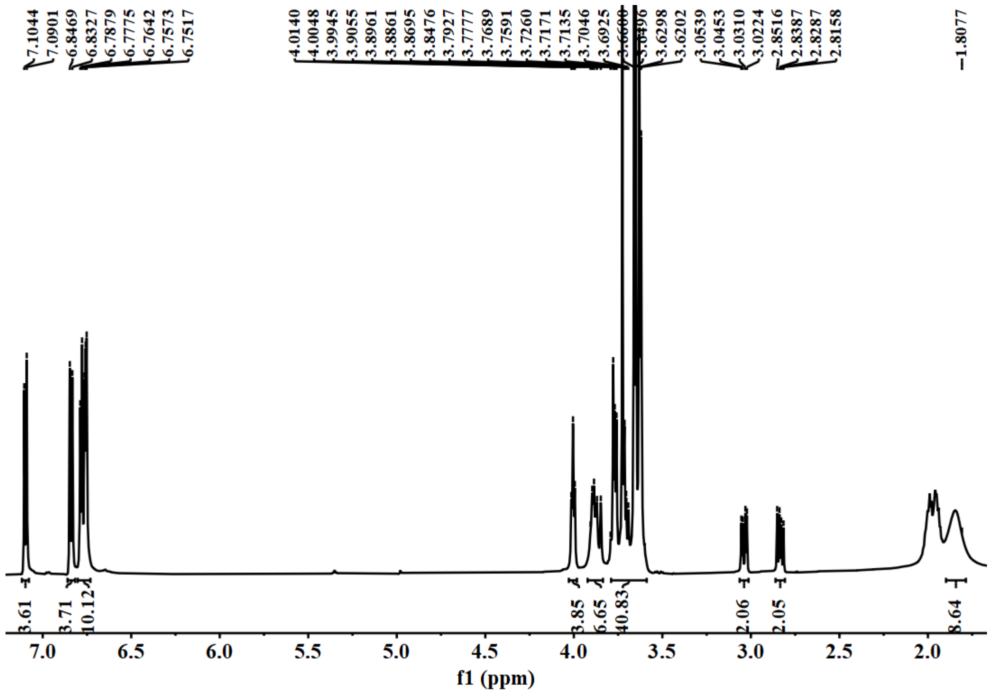

Figure S10. ^{1}H NMR spectrum of L-**P5** in $CDCl_3$.

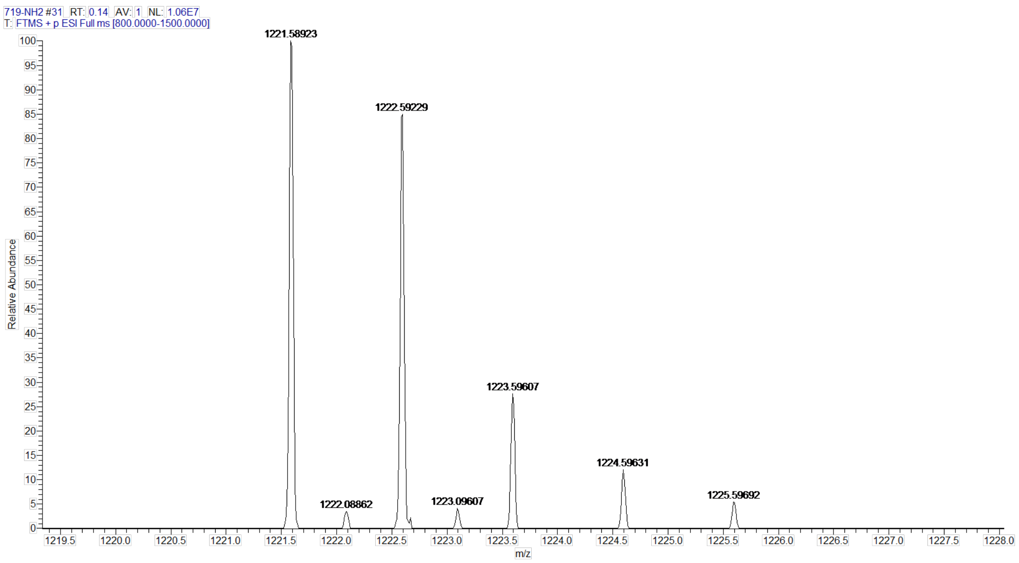

Figure S11. ESI-MS spectrum of L-**P5**.

3. Synthesis of A

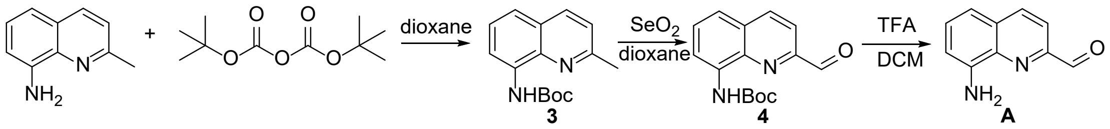

Scheme *S*2. Synthetic route of **A**

8-Aminoquinaldine (0.50g, 3.16 mmol) was stirred with *di*-t-butyldicarbonate (1.72g, 7.90 mmol) in 20 mL of 1,4-dioxane at 85°C for two days. The solvent was removed under reduced pressure and the residue was purified by column chromatography on silica gel using dichloromethane/petroleum ether (DCM/PE =1/1, v/v) as eluent to give 0.57g (70%) of **3** as white crystals. melting point: 72–73°C; IR (KBr) cm^{-1}: 3367.35 (N-H), 1718.37 (C=O), 1606.12 (C=C), 1518.37, 1489.80 (ArC=C), 1569.39 (C=N), 1014.29 (C-O); ^{1}H NMR (500 MHz, Chloroform-d) δ 9.05 (s, 1H), 8.37 (d, J = 7.6 Hz, 1H), 8.00 (d, J = 8.4 Hz, 1H), 7.43 (t,

J = 7.9 Hz, 1H), 7.36 (dd, J = 8.2, 1.3 Hz, 1H), 7.29 (d, J = 8.4 Hz, 1H), 2.73 (s, 3H), 1.59 (s, 9H). ^{13}C NMR (126 MHz, Chloroform-d) δ 156.97, 152.99, 137.58, 136.31, 134.57, 126.21 (d, J = 17.2 Hz), 122.33, 119.93, 114.35, 80.34, 28.45, 27.43, 25.20.

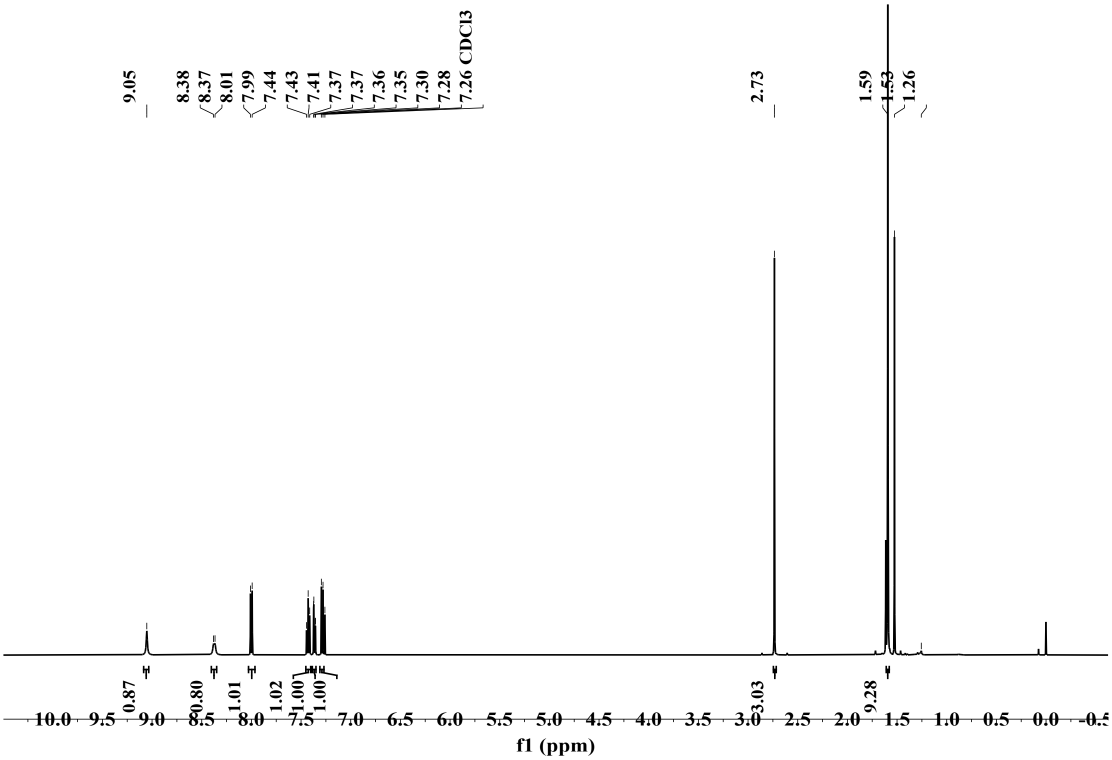

Figure S12. ^{1}H NMR spectrum of **3** in $CDCl_3$.

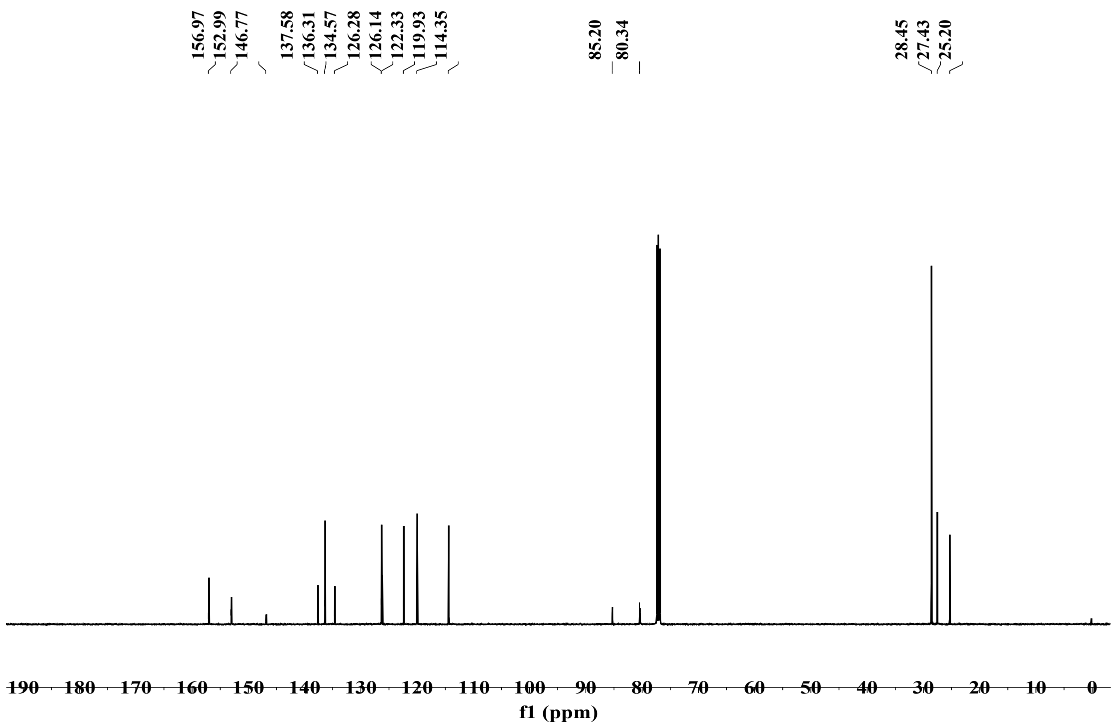

Figure S13. ^{13}C NMR spectrum of **3** in $CDCl_3$.

To a stirred suspension of freshly sublimed SeO_2 (429.58mg, 3.87 mmol) in 20 ml of 1,4-dioxane at 50–55°C was added a solution of **3** (0.5g,1.94 mmol) in 50 mL of 1,4-dioxane during the course of 3h. The mixture was heated to 80–85°C overnight, filtered, and the dioxane was removed under reduced pressure. The residue was purified by column chromatography on silica gel with dichloromethane/petroleum ether (DCM/PE =1/1, v/v) as eluent to give 0.40 g (75%) of as yellow crystals **4**, melting point: 129–131°C; IR(KBr)cm^{-1}: 3379.78(N-H),1704.08 (C=O), 1616.50 (C=C), 1536.91, 1487.93 (Ar-C=C), 1569.56 (C=N), 1004.25(C-O); ^{1}H NMR (600 MHz, Chloroform-d) δ 10.26 (s, 1H), 9.00 (s,

1H), 8.52 (d, J = 7.8 Hz, 1H), 8.29 (d, J = 8.4 Hz, 1H), 8.05 (d, J = 8.4 Hz, 1H), 7.66 (t, J = 7.8, Hz, 1H), 7.51 (d, J = 8.3, Hz, 1H), 1.64 (s, 9H). ^{13}C NMR (151 MHz, Chloroform-d) δ 193.17, 152.69, 150.23, 137.70, 137.64, 136.18, 130.50, 130.07, 120.07, 117.68, 115.35, 81.06, 29.67, 28.40, 27.43.

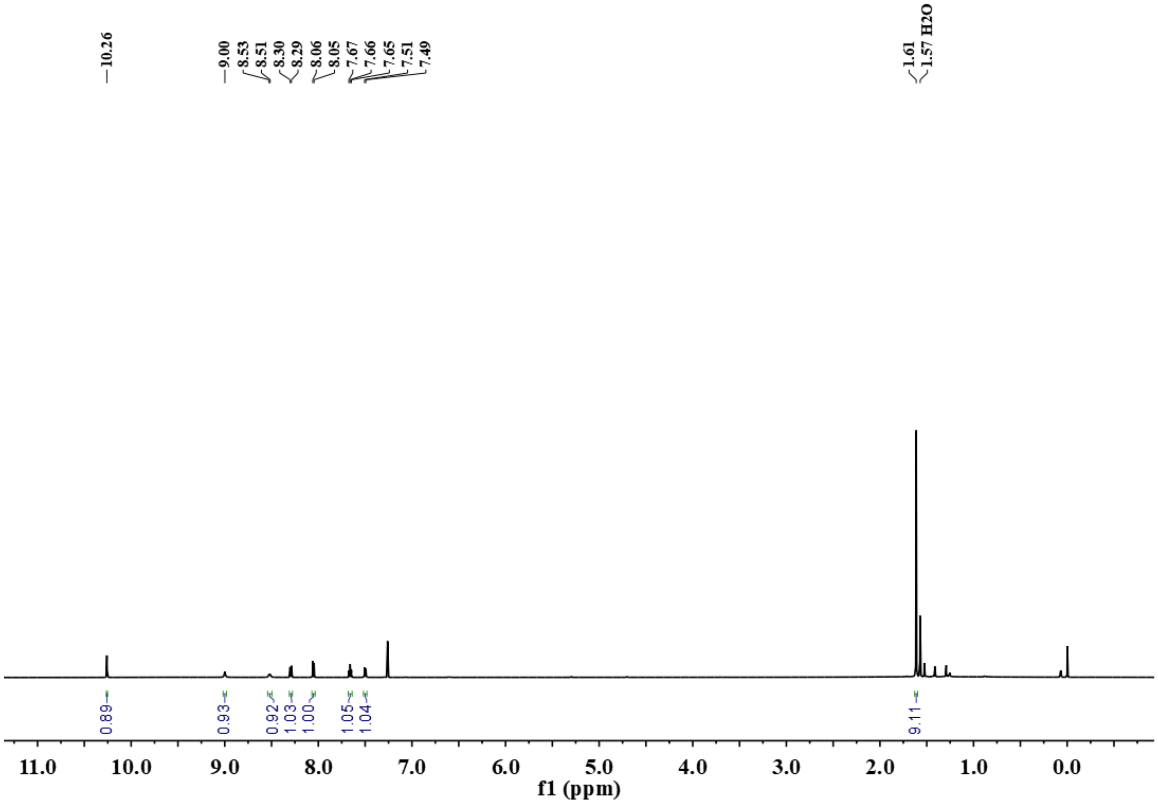

Figure S14. ^{1}H NMR spectrum of **4.**

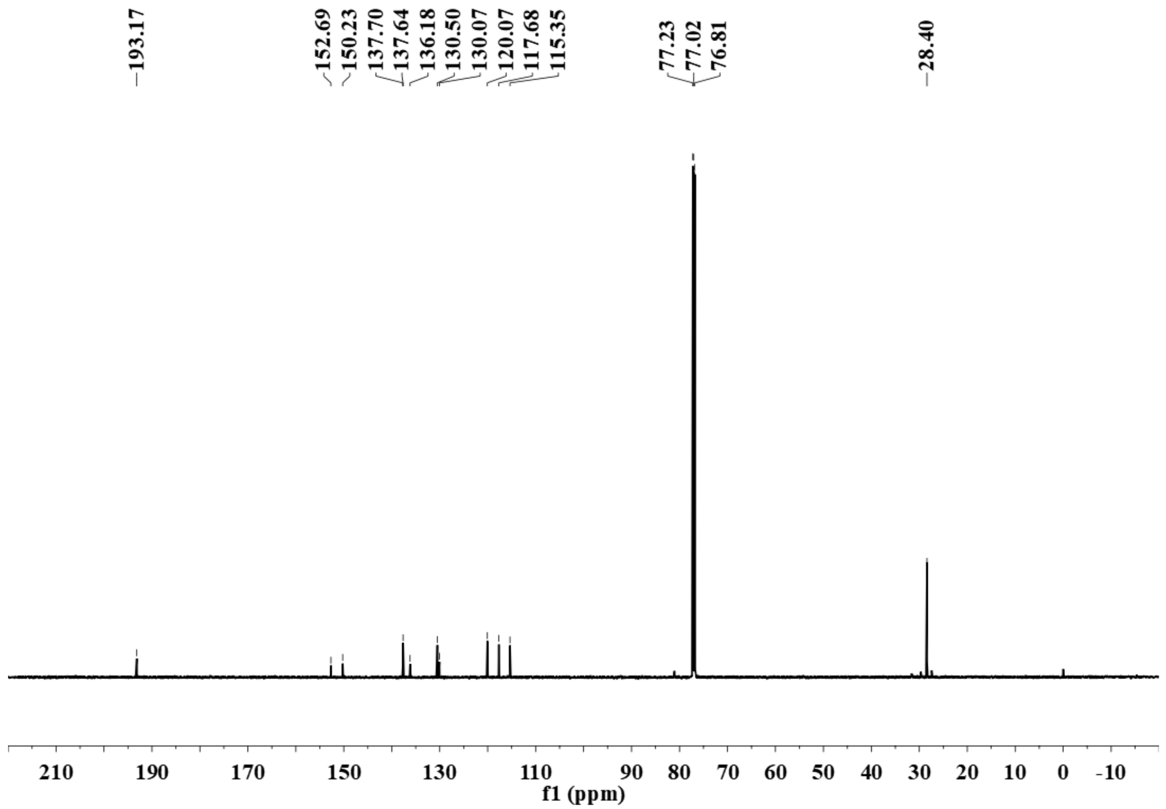

Figure S15. ^{13}C NMR spectrum of **4.**

4 (0.3g,1.10 mmol) was dissolved in a mixture of dichloromethane and trifluoroacetic acid (3:1,v/v,30.0 mL). The reaction mixture was left to stir at room temperature for 45 min resulting in a dark red solution. This mixture was poured over ice and neutralised with sodium bicarbonate, yielding an orange solution at pH =7. The organic phase was extracted with dichloromethane and washed with H_2O (2×50 ml) and subsequently dried over $MgSO_4$ and concentrated in vacuo. This yielded was 141.46 mg (78%) dark orange crystalline solid **A**. IR(KBr) cm^{-1}: 3479.59, 3373.47(N-H), 1716.33(C=O), 1608.16 (C=C), 1526.53, 1475.51 (Ar-C=C), 1571.43 (C=N); ^{1}H NMR (600 MHz, Chloroform-d) δ 10.20 (s, J = 0.9 Hz, 1H), 8.20

(d, J = 8.4, 0.9 Hz, 1H), 7.98 (d, J = 8.4 Hz, 1H), 7.47 (t, J = 7.8 Hz, 1H), 7.19 (d, J = 8.1, 1.2 Hz, 1H), 6.98 (d, J = 7.6, 1.2 Hz, 1H), 5.20 (s, 2H).

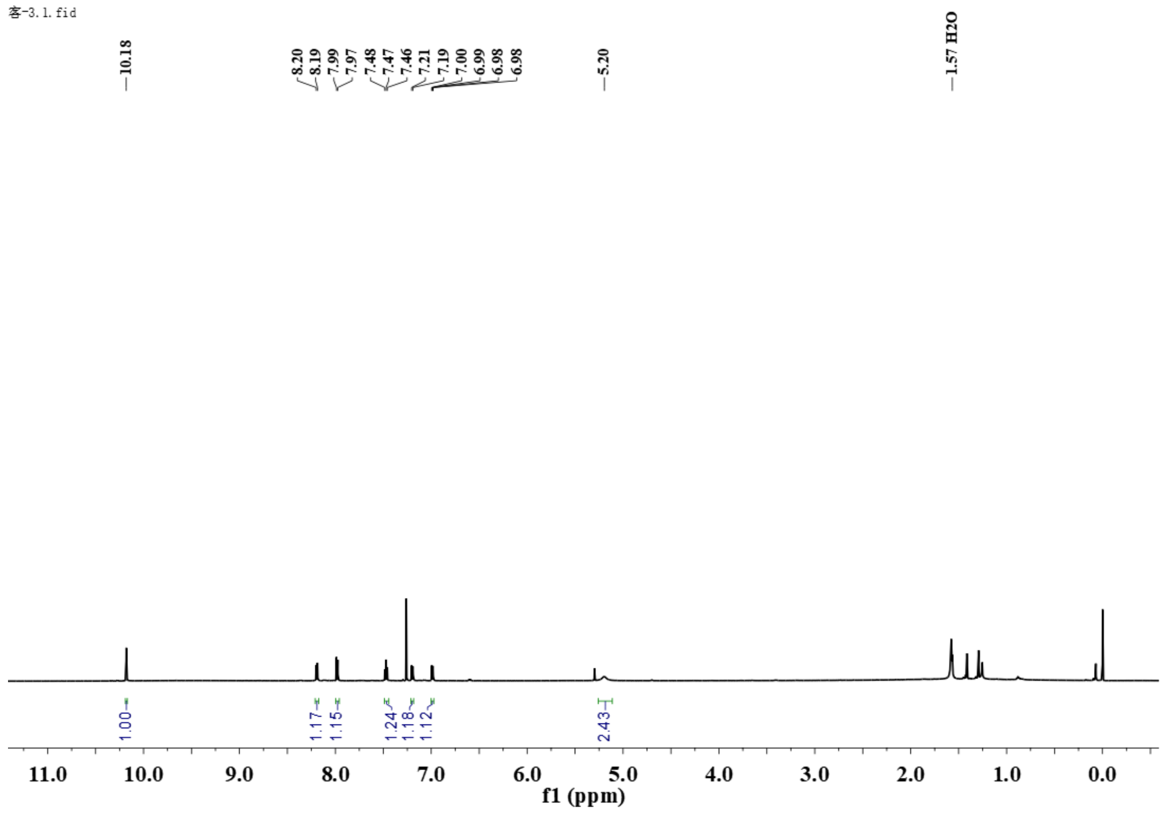

Figure S16. ^{1}H NMR spectrum of **A**.

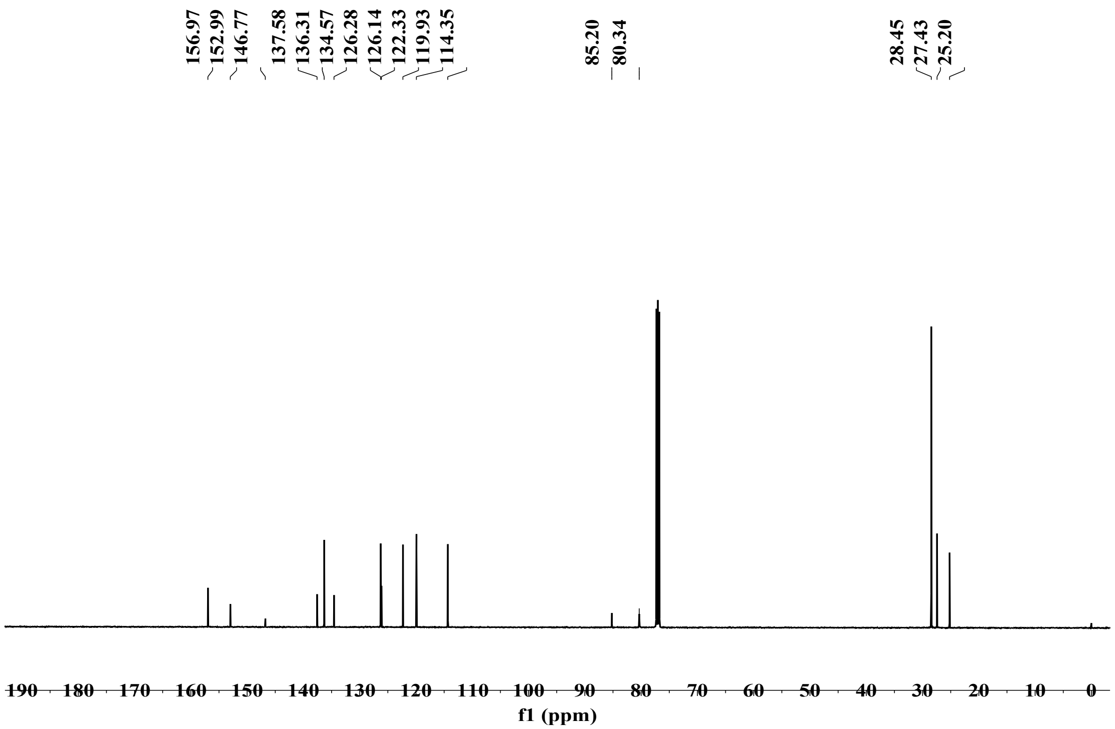

Figure S17. ^{13}C NMR spectrum of **A**.

4. Synthesis of Cu(I)

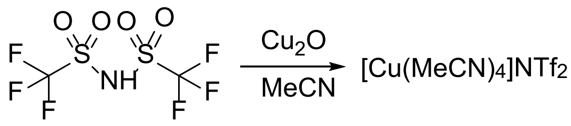

Scheme *S*3. Synthetic route of **Cu(I)**

Bis(trifluoromethylsulfonyl)imide (1.6g, 5.7mmol) was weighed into a Schlenk tube under an inert atmosphere of nitrogen. To this was added CH_3CN (10 ml) and copper(I) oxide (Cu_2O) powder (0.8g, 2.8mmol). The resultant slurry was thoroughly degassed, sealed under nitrogen and stirred for 15 h. The reaction mixture was then filtered through Celite. The filtrate was then dried, dissolved in CH_2Cl_2 and crystallized by diffusion of diethylether to afford a colorless solid

[Cu(MeCN)$_4$]NTf$_2$ **Cu(I)**(0.5 g, 80%). ^{1}H NMR (600 MHz, 298 K, CDCl$_3$): δ =2.17 (s, 12H; CH3CN); ^{19}F NMR (600 MHz, 298 K, CDCl$_3$): δ = –78.8 ppm.

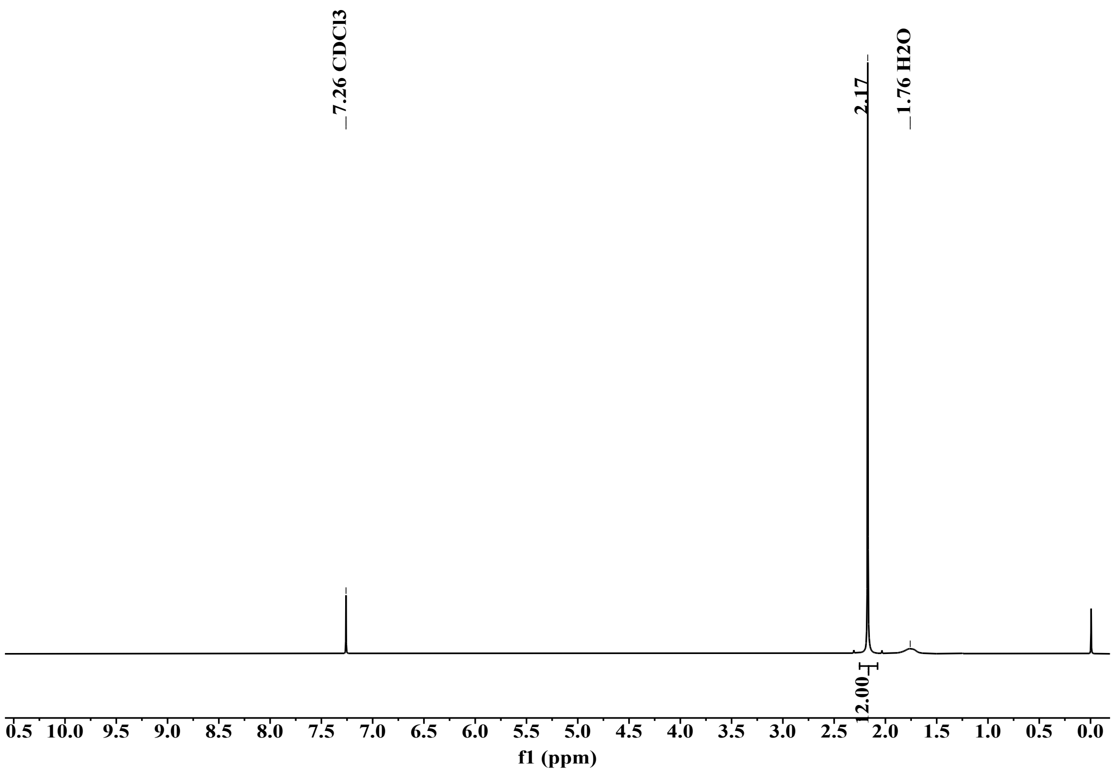

Figure S18. ^{1}H NMR spectrum of **[Cu]**.

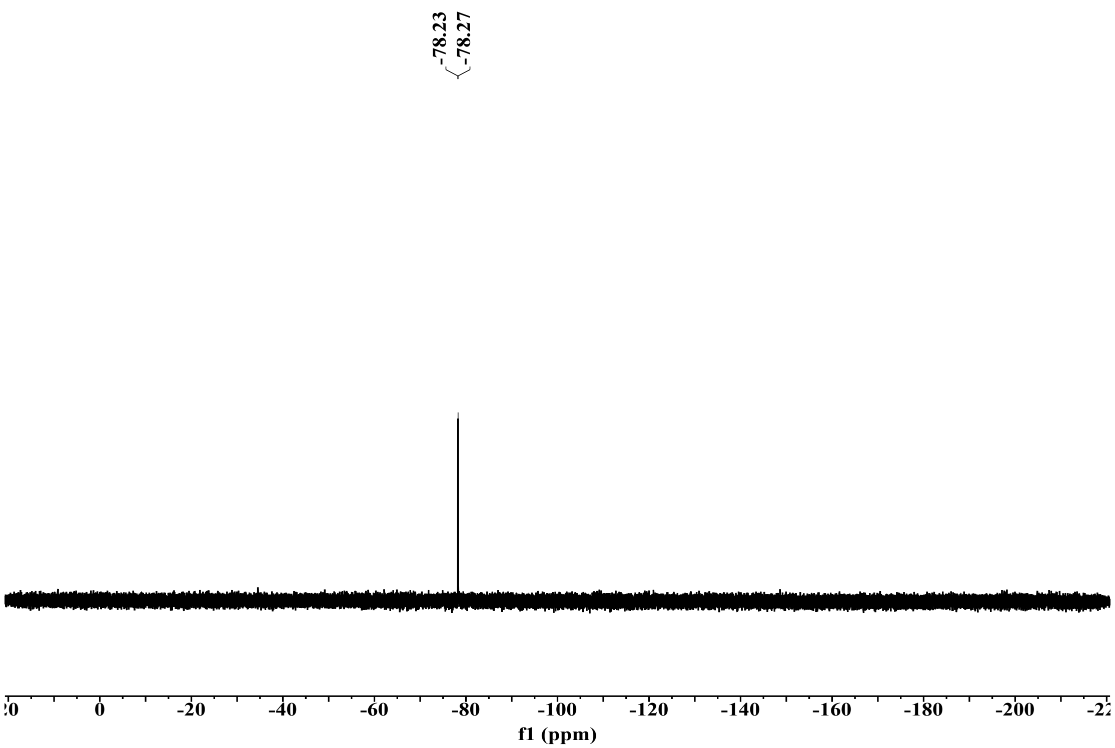

Figure S19. ^{19}F NMR spectrum of **[Cu]**.

5. Typical self-assembly procedure

Under N_2 atmosphere, monomer A (5 mg, 0.030 mmol, 16 eq), Cu(I) (9.68 mg, 0.015 mmol, 9 eq), and P5 (4.88 mg, 10 mg/mL dissolved in CH_3CN, 3.75 umol, 2 eq) were added to dry, degassed CH_3CN (0.5 mL) to give a brown solution, which was stirred at room temperature for 48 h. The solvent was evaporated to give a dark brown solid. The solution was stored under N_2 atmosphere until further experiments/analysis were required.

Supramolecular self-assembly systems with different molar ratio of P5/A/Cu(I) were as follows:

Entry	Molar ratio(equiv) P5:A:Cu(I)	Supramolecular self-assembly systems

1	1： 16： 9	(*P*)-**Helix1**
2		(*M*)-**Helix1**
3	2： 16： 9	(*P*)-**Helix2**
4		(*M*)-**Helix2**
5	4： 16： 9	(*P*)-**Helix3**
6		(*M*)-**Helix3**
7	1： 8： 9	(*P*)-**Helix4**
8		(*M*)-**Helix4**
9	2： 8： 9	(*P*)-**Helix5**
10		(*M*)**-Helix5**
11	4： 8： 9	(*P*)-**Helix6**
12		(*M*)-**Helix6**

The supramolecular self-assemblies prepared by chiral induction with D-**P5** were named (*P*)-**Helix**, while those induced with L-**P5** chirality were (*M*)-**Helix**。

6. CD spectra

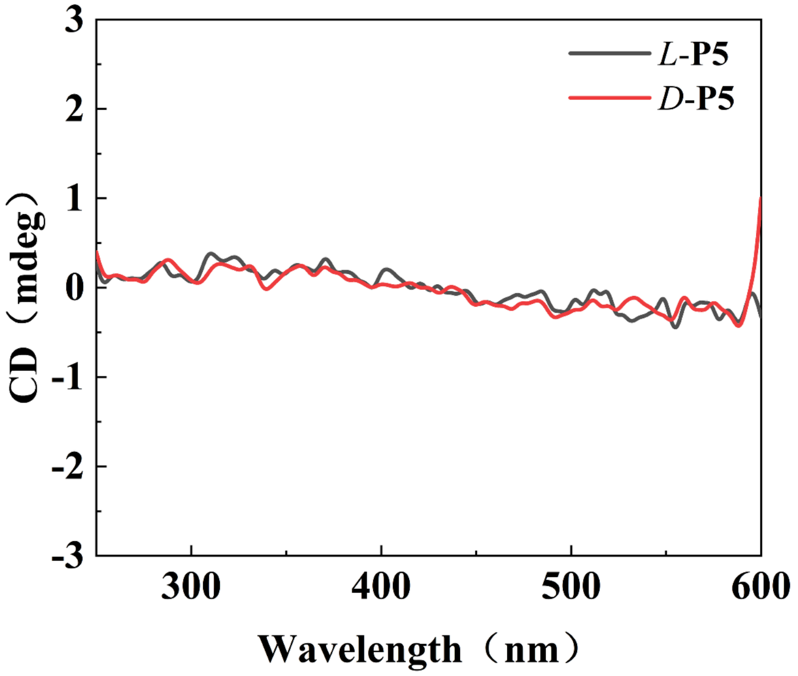

Figure S20. CD spectra of different ratios of D/L-**P5** at c=1×10^{-4}mol/L in different solutions

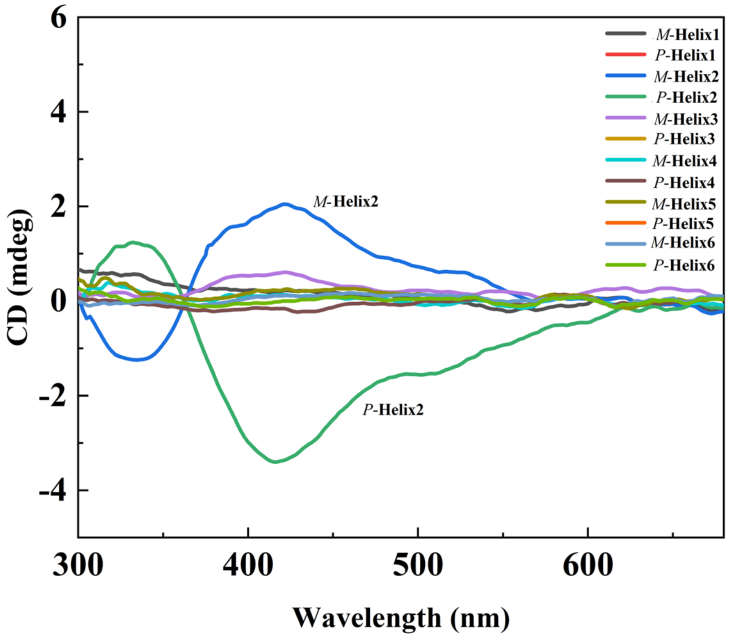

Figure S21. CD spectra of different ratios of (*P*)**-Helix2** and (*M*)**-Helix2** at c=1×10-

[4]mol/L in different solutions

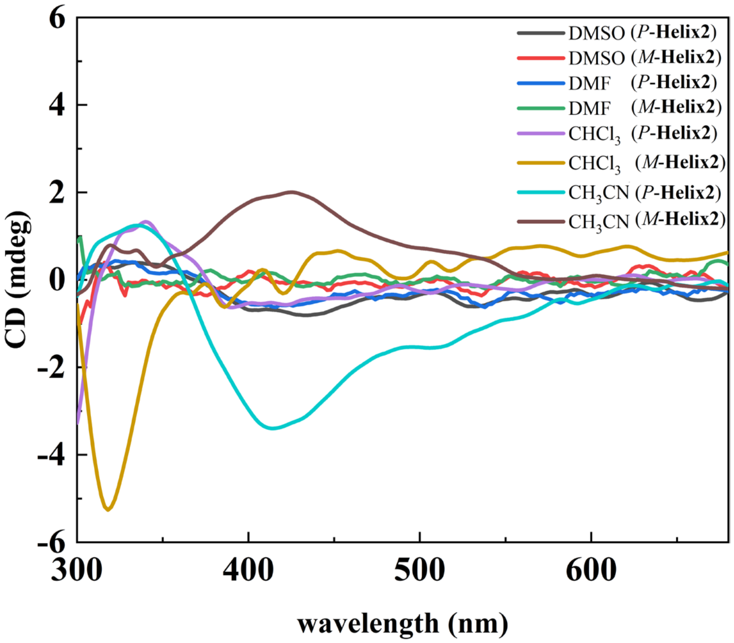

Figure S22. CD spectra of (*P*)**-Helix2** and (*M*)**-Helix2** at c=1×10^{-4} mol/L in different solutions.

7. Asymmetric catalytic reactions with supramolecular catalysts

Table *S*1. Effect of the reaction conditions on the cyano-trifluoromethylation reactions of olefins using (*M*)-Helix2 as catalyst [a].

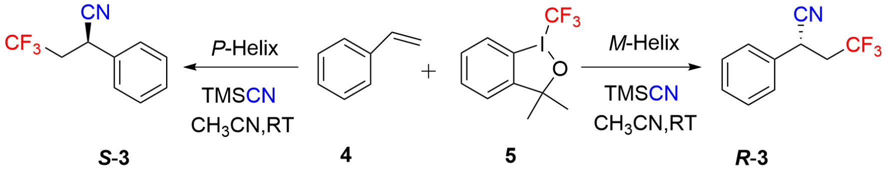

Entry	Catalyst（mol%）	Solvent	T (h)	T°C)	Yield (%) [b]

1	(*M*)-**Helix2**(5)	DMSO	12	60	trace
2	(*M*)-**Helix2**(5)	DMF	12	60	trace
3	(*M*)-**Helix2**(5)	$CHCl_3$	12	60	trace
4	(*M*)-**Helix2**(5)	CH_3CN	12	60	82
5	(*M*)-**Helix2**(5)	DMSO	12	r. t.	trace
6	(*M*)-**Helix2**(5)	DMF	12	r. t.	trace
7	(*M*)-**Helix2**(5)	$CHCl_3$	12	r. t.	trace
8	(*M*)-**Helix2**(5)	CH_3CN	12	r. t.	89
9	(*M*)-**Helix2**(10)	DMSO	12	60	trace
10	(*M*)-**Helix2**(10)	DMF	12	60	trace
11	(*M*)-**Helix2**(10)	$CHCl_3$	12	60	trace
12	(*M*)-**Helix2**(10)	CH_3CN	12	60	71
13	(*M*)-**Helix2**(10)	DMSO	12	r. t.	trace
14	(*M*)-**Helix2**(10)	DMF	12	r. t.	trace
15	(*M*)-**Helix2**(10)	$CHCl_3$	12	r. t.	trace
16	(*M*)-**Helix2**(10)	CH_3CN	12	r. t.	69

[a] Reaction conditions: 4 (0.1 mmol), Togni reagent 5 (0.12 mmol), TMSCN (0.15 mmol), solvent (2.0 mL)

[b] NMR yield, determined by 19F NMR spectroscopy using benzotrifluorideas an internal standard

Table *S*2. the self-assembled supramolecular catalysts with different copper ratios were investigated for catalytic yields and enantioselectivitie.

Entry	Molar ratio(equiv) **P5**: **A**:Cu(I)	Supramolecular catalyst	Yield (%)	ee(%):
1	1:16:9	(*P*)-**Helix1**	39	85 (R)
2		(*M*)-**Helix1**	84	12 (S)
3	2:16:9	(*P*)-**Helix2**	35	28 (R)
4		(*M*)-**Helix2**	89	3 (S)
5	4:16:9	(*P*)-**Helix3**	20	21 (R)
6		(*M*)-**Helix3**	70	59 (S)
7	1:8:9	(*P*)-**Helix4**	29	-
8		(*M*)-**Helix4**	33	-
9	2:8:9	(*P*)-**Helix5**	16	-
10		(*M*)-**Helix5**	40	-
11	4:8:9	(*P*)-**Helix6**	13	-
12		(*M*)-**Helix6**	27	-
13	0:2:1	***Rac*-2**	85	<2% (R)

8. HPLC of the Products

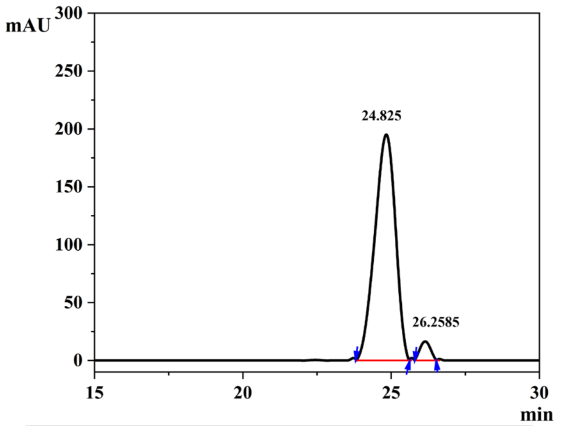

Peak#	Ret. Time	Area	Height	Area%	Height%
1	24.825	100.95	194.46	91.75	92.25
2	26.2585	9.08	16.34	8.25	7.75
Total		110.03	210.8	100	100

Figure S23. HPLC of products synthesized over (*P*)-**Helix1** catalyst.

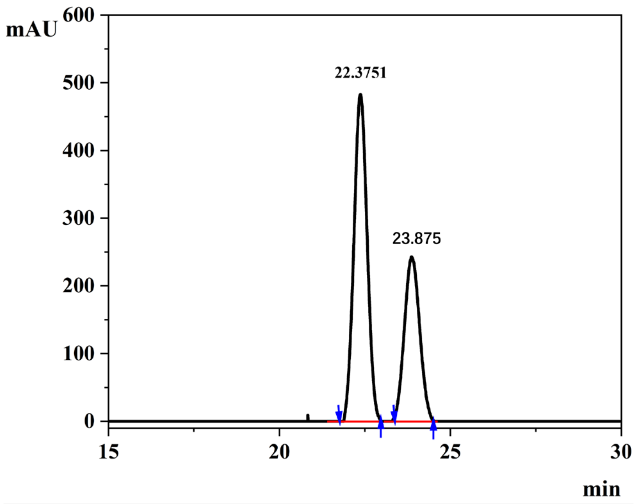

Peak#	Ret. Time	Area	Height	Area%	Height%
1	22.3751	81.174	481.16	63.79	66.70
2	23.875	46.066	240.17	36.21	33.3
Total		127.24	721.33	100	100

Figure S24. HPLC of products synthesized over (*P*)-**Helix2** catalyst.

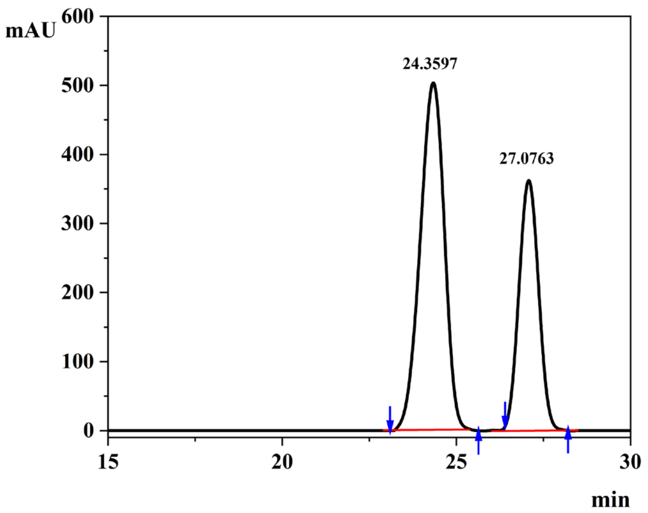

Peak#	Ret. Time	Area	Height	Area#	Height#
1	24.3597	154.354	527.06	60.43	61.20
2	27.0763	101.076	334.15	39.57	38.80
Total		255.43	861.21	100	100

Figure S25. HPLC of products synthesized over (*P*)-**Helix3** catalyst.

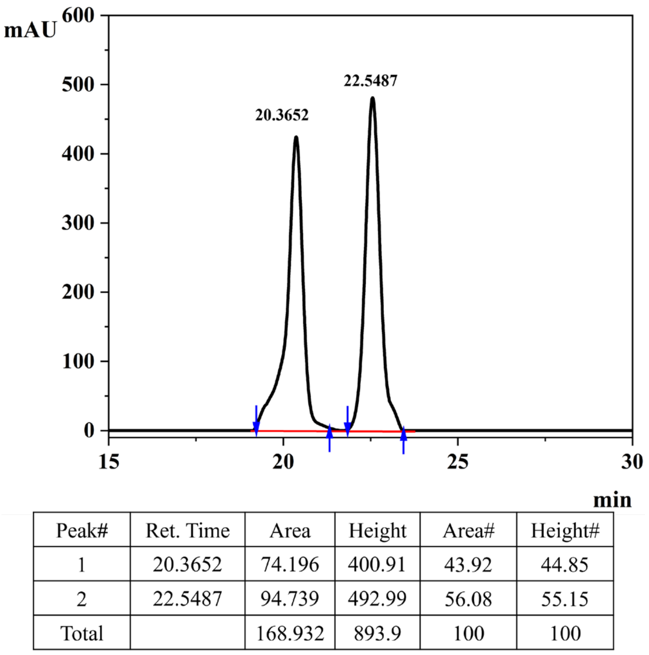

Peak#	Ret. Time	Area	Height	Area#	Height#
1	20.3652	74.196	400.91	43.92	44.85
2	22.5487	94.739	492.99	56.08	55.15
Total		168.932	893.9	100	100

Figure S26. HPLC of products synthesized over (*M*)-**Helix1** catalyst.

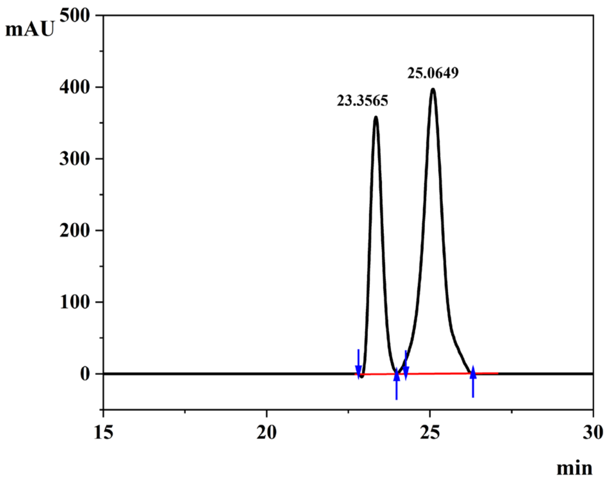

Peak#	Ret. Time	Area	Height	Area#	Height#
1	23.3565	143	367.90	48.58	49.23
2	25.0649	151.58	379.41	51.46	50.77
Total		294.58	747.31	100	100

Figure S27. HPLC of products synthesized over (*M*)-**Helix2** catalyst.

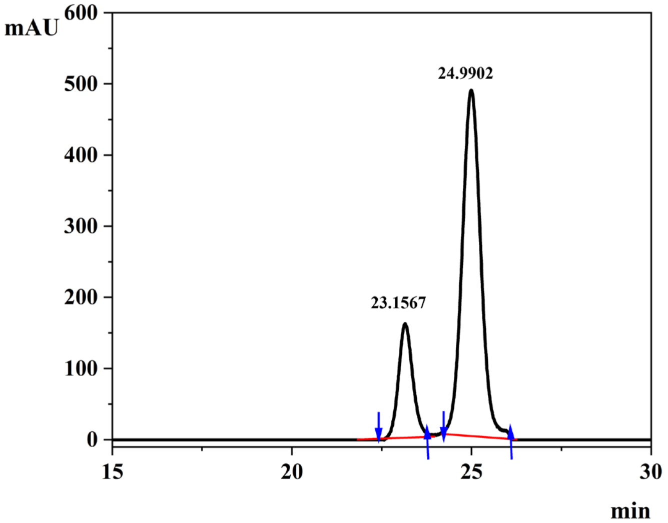

Peak#	Ret. Time	Area	Height	Area#	Height#
1	23.1567	34.413	140.944	20.63	21.78
2	24.9902	132.361	506.181	79.37	78.22
Total		166.774	647.125	100	100

Figure S28. HPLC of products synthesized over (*M*)-**Helix3** catalyst.

9.AFM of Helix

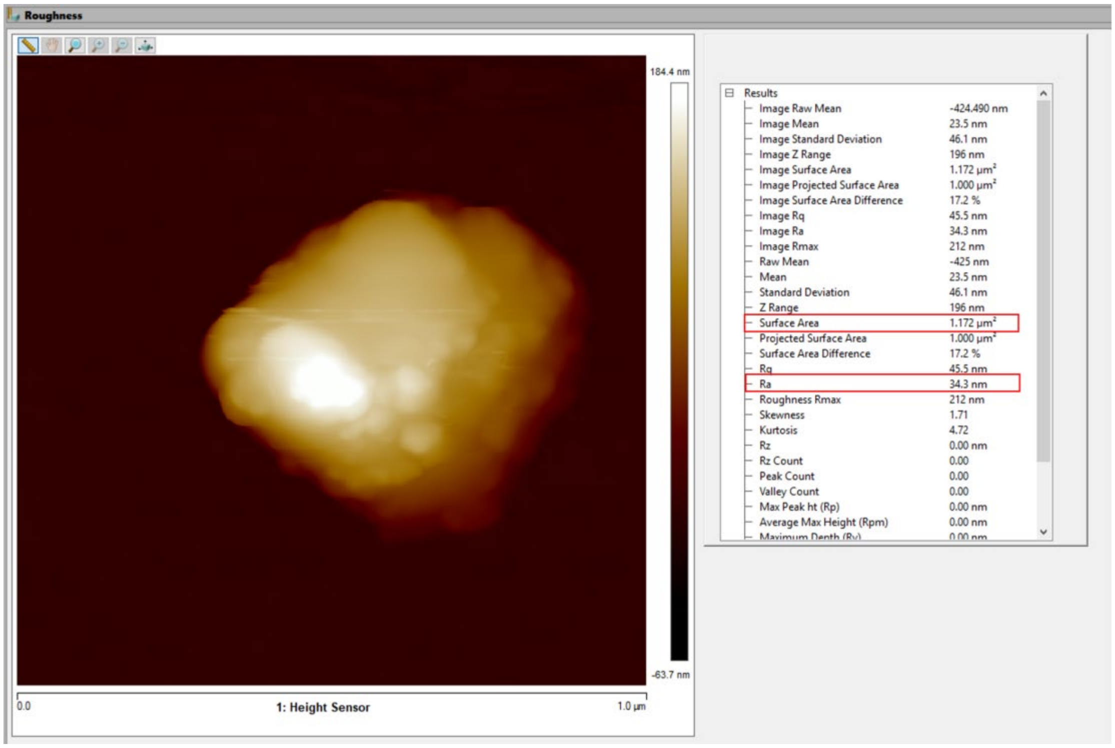

Figure S29. AFM of (*P*)-**Helix**.

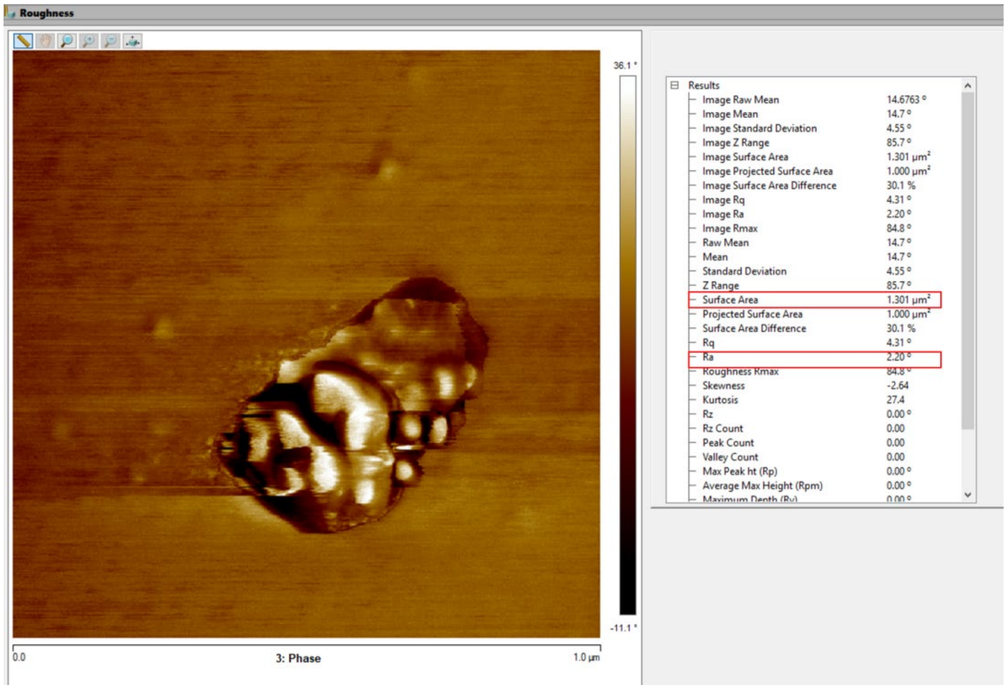

Figure S30. AFM of (*M*)-***Helix*.**

10. References

[1] Liu, L. Z.; Cao, D. R.; Jin, Y.; Tao, H. Q.; Kou, Y. H.; Meier, H., Efficient synthesis of copillar[5]arenes and their host-guest properties with dibromoalkanes. *Org Biomol Chem* **2011,** *9* (20), 7007-7010.
